# Screening for Higher Grain Yield and Biomass among Sixty Bread Wheat Genotypes Grown under Elevated CO_2_ and High-Temperature Conditions

**DOI:** 10.3390/plants10081596

**Published:** 2021-08-03

**Authors:** Emilio L. Marcos-Barbero, Pilar Pérez, Rafael Martínez-Carrasco, Juan B. Arellano, Rosa Morcuende

**Affiliations:** Institute of Natural Resources and Agrobiology of Salamanca (IRNASA), Consejo Superior de Investigaciones Científicas (CSIC), 37008 Salamanca, Spain; emiliol.marcos@irnasa.csic.es (E.L.M.-B.); pili2013@gmail.com (P.P.); rafael.mcarrasco@gmail.com (R.M.-C.); juan.arellano@irnasa.csic.es (J.B.A.)

**Keywords:** climate change, elevated CO_2_, high temperature, grain yield, biomass, bread wheat, genotypes

## Abstract

Global warming will inevitably affect crop development and productivity, increasing uncertainty regarding food production. The exploitation of genotypic variability can be a promising approach for selecting improved crop varieties that can counteract the adverse effects of future climate change. We investigated the natural variation in yield performance under combined elevated CO_2_ and high-temperature conditions in a set of 60 bread wheat genotypes (59 of the 8TH HTWSN CIMMYT collection and Gazul). Plant height, biomass production, yield components and phenological traits were assessed. Large variations in the selected traits were observed across genotypes. The CIMMYT genotypes showed higher biomass and grain yield when compared to Gazul, indicating that the former performed better than the latter under the studied environmental conditions. Principal component and hierarchical clustering analyses revealed that the 60 wheat genotypes employed different strategies to achieve final grain yield, highlighting that the genotypes that can preferentially increase grain and ear numbers per plant will display better yield responses under combined elevated levels of CO_2_ and temperature. This study demonstrates the success of the breeding programs under warmer temperatures and the plants’ capacity to respond to the concurrence of certain environmental factors, opening new opportunities for the selection of widely adapted climate-resilient wheat genotypes.

## 1. Introduction

Climate change is considered to have detrimental effects on global food security by modifying the environmental conditions for agricultural production. The progressive increase in atmospheric CO_2_ concentration and other greenhouse gases due to anthropogenic activities, such as continuous deforestation and the excessive use of fossil fuels, is the main driving force for global warming [1]. The atmospheric concentrations of CO_2_ have risen since preindustrial times, and currently exceed 410 ppm [2]; this is expected to continue rising over this century, to reach levels close to 1000 ppm by 2100. Simultaneously, the global mean surface air temperature is predicted to rise by an average of 2.6–4.8 °C throughout this century, which may result in an increase in the incidence of other weather events with negative consequences for crop productivity [3]. Therefore, climate variability is threatening the current food system because crop growth and production are markedly affected by both the atmospheric concentration of CO_2_ and air temperature [4].

Wheat is among the three main staple crops worldwide, covering more than 20% of the total calorie intake by the worldwide human population, and an equivalent percentage of proteins for approximately 2.5 billion people [5]. Bread wheat (*Triticum aestivum* L.) is extensively cultivated in Mediterranean regions, where the elevated temperature is likely the major yield-limiting factor [6].

One of the main effects of elevated atmospheric CO_2_ on C3 crops such as wheat is an increase in the photosynthesis rate, which may thereby enhance plant growth and consequently increase grain yield. However, the initial stimulation of photosynthesis, as induced by elevated CO_2_, cannot be sustained over prolonged exposure to high CO_2_ concentrations, and it also results in a down-regulation of the photosynthetic capacity [7,8]. This acclimation of photosynthesis to elevated CO_2_ is often found in wheat [9,10,11,12,13,14] and is associated with enhanced leaf carbohydrate content and decreased N concentration [14,15,16,17], yielding grains with lower N and protein concentration [18,19]. The acclimation of photosynthesis is particularly common in plants grown in pots with a restricted root system [20] or with low nutrient availability [12,21,22], and often reflects the plant’s sink strength [23]. Environmental or genetic factors limiting the development of sink strength make plants susceptible to a greater photosynthetic acclimation, and diminish the stimulation of photosynthesis by CO_2_ enrichment [7,8]. Thus, source–sink relationships have been proposed as playing a key role in the regulation of plant growth and yield response to elevated atmospheric CO_2_ [23,24]. In line with this suggestion, greater growth and yield response to elevated CO_2_ has been found in some older wheat cultivars when compared to modern ones, a finding which seems to be associated with the capacity of the older cultivars to produce further tillers functioning as vegetative sinks for the allocation of excess carbohydrates [25,26]. Increased tillering is not the only sink of relevance for wheat crops to deal with excess carbohydrates. The translocation of carbohydrates into grains during the reproductive growth stages is entailed in determining grain yield, and the sink strength of the maturing ears and grains becomes crucial [14,27,28]. 

The world is also facing a gradual rise in temperature, and most of the worldwide wheat-growing regions are undergoing temperature increases greater than are optimal, leading to alterations in plant growth, development, physiological processes, and yield [29,30]. The evaluation of the impact of global warming on six major food crops, performed by Lobell and Field (2007) [31], showed yield losses of about 40 million tonnes per year as a whole for wheat, maize and barley from 1981 to 2002, where wheat accounts for nearly half of these shortfalls (19 mt/year). This finding highlights that wheat is highly sensitive to high temperatures; the occurrence of elevated temperatures at any developmental stage can affect grain yield, although they are particularly critical at the reproductive stage and cause considerable yield losses [32,33]. Bergkamp et al. (2018) [34] reported that heat stress (35 °C) after anthesis decreased the grain-filling period and restricted the deposition of resources to grains, which led to a substantial decrease in wheat productivity by 6–51% when plants were grown in controlled conditions and by 2–27% under field conditions. Such yield losses have been linked to the direct impact of temperature on grain number and grain weight [35,36]. Further works using global- and/or country-based models showed that an increase in 1 °C above the average global surface temperatures could result in a worldwide decline in wheat yields from 4.1% to 6.4% [37,38]. Thus, the prediction of the potential impact of global warming on crop productivity is complicated because CO_2_ enrichment could result in yield stimulation; and, for several crops, including wheat, this could ameliorate some of the adverse effects of higher temperatures on yields [39]. In line with this, Fitzgerald et al. (2016) [40] observed that growth under elevated atmospheric CO_2_ enhanced biomass and yield in bread wheat grown in FACE facilities and, when combined with higher temperatures, it counteracted the negative impacts of temperature on grain yield.

Worldwide demand for wheat is projected to continue growing, whereas the current increase in wheat productivity is only 1.1% per year, while there is simply no increase in some regions [6]. Consequently, the actual wheat yield gain per year is insufficient to meet the demand for food from the increasing world population. Improving yield and its sustainability at a time of unprecedented climatic variability is becoming more and more urgent as the global population increases. To tackle these limitations, improved crop varieties will be required to guarantee food security. The exploitation of genetic variations in wheat responses to future climatic conditions might be a useful approach. Nevertheless, as mentioned above, there is a wealth of studies dealing with the present and predicted impact of elevated atmospheric CO_2_ concentrations and temperature on the productivity of wheat under controlled and field conditions, but most of them have been carried out without considering simultaneous increases in atmospheric CO_2_ and temperature, and are conducted with a single genotype or a limited range of crop germplasm [41]. The aim of this work was to assess the natural existing variation in wheat yield performance in response to combined elevated CO_2_ and high temperature. We used a wide set of 60 bread wheat genotypes, including 59 genotypes of the CIMMYT heat-tolerant wheat screening nursery (8TH HTWSN) previously selected for high performance under warmer temperatures, together with the Gazul genotype, with high adaptability to the Mediterranean climate of the Salamanca region (Spain). All genotypes were grown until physiological maturity in controlled climate chambers that simulate the predicted global warming by the end of this century, in the region of Salamanca; the final biomass and grain yield components were determined. The results of our study will enable better planning for the selection of cultivars with improved adaptation to future climate change.

## 2. Results

### 2.1. Wheat Production and Grain Yield Components 

Table 1 summarizes a list of descriptive statistics for wheat production and grain yield components, in response to the combined elevated atmospheric CO_2_ and high temperatures across a population of 60 wheat genotypes, cultivated in climate chambers. Among genotypes, grain yield ranged from 4.63 to 10.70 g per plant, although 50% of the genotypes produced between 6.96 and 8.43 g per plant. The mean grain yield was 7.66 ± 1.12 g per plant, with genotypes 150, 74, 23, 8, and 76 as the lowest yielding genotypes, and 43, 94, 95, 61 and 41 as the highest yielding genotypes. The aboveground, stalk and chaff biomasses also ranged between 13.13−25.50, 5.99−12.21 and 1.54−4.42 g per plant, respectively, with 19.16 ± 2.51, 8.78 ± 1.20 and 2.72 ± 0.55 g per plant on average for the whole population. The mean plant height was 86.82 ± 4.88 cm per plant, ranging from 76.35 to 105.30 cm per plant. Grain number per ear (GNE) and per plant varied from 19.4 and 116.39 grains to 54.02 and 315.33 grains, respectively, with an average of 34.99 ± 5.63 grains per ear and 197.01 ± 36.05 grains per plant. The number of ears per plant ranged between 3.20 and 8.20, with a mean value of 5.67 ± 0.84 ears per plant. Grain yield per ear (GYE) varied from 0.89 to 1.96, with 1.36 ± 0.19 g per ear on average. The mean harvest index (HI) was 0.40 ± 0.03, ranging from 0.26 to 0.46. Skewness and kurtosis of variables in the population were generally located around zero, suggesting symmetry and tail weight similar to that of normal distribution. However, the skewness and kurtosis of HI accounted for −0.77 and 2.68, indicating a non-normal distribution with moderate left-skewed asymmetry. Statistically significant differences among genotypes were found for the studied traits.

### 2.2. Comparison between the CIMMYT Population and Gazul 

**Figure 1:**
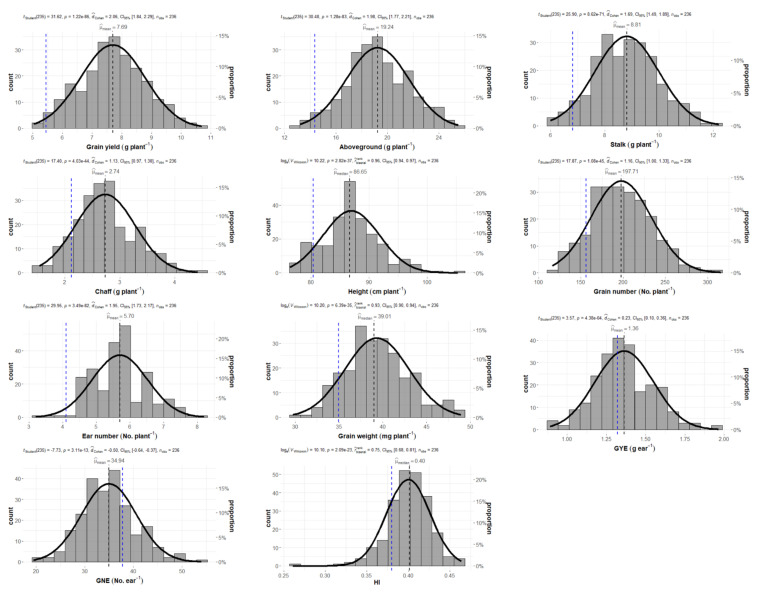
Histograms with a one-sample test of wheat production and grain yield components for 59 wheat genotypes, from CIMMYT grown under elevated CO_2_ and high-temperature conditions. *GNE*: grain number ear^−1^; *GYE*: grain yield ear^−1^; *HI*: harvest index. Each plot shows the distribution of frequencies with a superimposed normal curve for the selected trait among a population of 59 wheat genotypes belonging to CIMMYT and their four replicates (*n*_obs_ = 236). Statistics (*t_student_* and *V_Wilcoxon_*), *p*-value (*p*) and the size of the effect (*d_Cohen_* and *r_Wilcoxon_*) with confidence intervals (CI) for a one-sample test are added. The black dotted line represents the mean or median of population, according to the test employed (Student’s *t*-test or Wilcoxon test). The blue dotted line represents the mean value for the four replicates (*n* = 4) of genotype 150 (Gazul). Differences were considered statistically significant at *p* < 0.05.

The mean responses of the genotype 150 (Gazul) for wheat production and grain yield traits were compared with a population of 59 selected wheat genotypes belonging to the CIMMYT 8TH HTWSN collection (hereafter called the CIMMYT population (Figure 1)). The one-sample test for the comparison of the mean responses showed significant differences between Gazul and the CIMMYT population for all the traits under study. Gazul exhibited lower aboveground, chaff and stalk biomasses, grain yield, grain and ear numbers per plant, and lower GYE and HI, but a greater GNE than the CIMMYT population. The effect size was considered as large for almost all the variables, with the exception of the effect size for GYE and GNE, which were considered as small and medium-size, respectively.

### 2.3. Correlation Network and Coefficient Matrix

**Figure 2:**
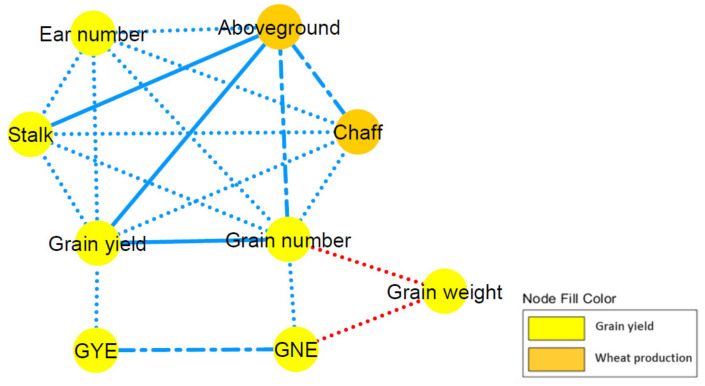
Correlation network for the wheat production and grain yield components of sixty wheat genotypes grown under elevated CO_2_ and high-temperature conditions. *GNE*: grain number ear^−1^; *GYE*: grain yield ear^−1^. The different traits (nodes) were classified by colours according to their grain yield or wheat production nature (see legend). Edges stand for a Spearman’s correlation *r* ≥ |0.45|, split up as dot (|0.65| > *r* ≥ |0.45|), dash and dot (|0.85| > *r* ≥ |0.65|) or solid (|1| > *r* ≥ |0.85|) line types. Blue edges indicate a positive correlation, whereas red lines implicate negative correlation.

To study the relationship among the traits under study in the population of the 60 wheat genotypes, both a correlation matrix and a correlation network were performed (Figure 2; Table S1). Positive correlations were found among the aboveground, stalk, chaff, grain yield, grain number and ear number traits, with very high correlations of aboveground biomass to stalk biomass (0.90), grain yield (0.88), chaff biomass (0.77) and grain number (0.76), as well as between grain yield and grain number (0.85). GYE and GNE were also positively correlated with each other (0.78), and both were correlated, to a lesser extent (0.11−0.61), with aboveground, stalk and chaff biomasses, grain yield and grain number traits. Furthermore, they were also negatively correlated with ear numbers (−0.44 and −0.29, respectively). Grain weight showed no correlation with chaff biomass and very low negative correlation with HI, stalk and aboveground biomasses, grain yield and ear number (ranged from −0.02 to −0.16), but it was negatively correlated with GNE (−0.54) and grain number per plant (−0.58). HI showed positive correlations with grain yield, GYE, grain number and GNE, but negative correlations with plant height and stalk and chaff biomasses, all of them lower than |0.45|. Plant height was in turn positively correlated with grain weight and with stalk and aboveground biomasses (0.28−0.15).

### 2.4. Principal Component and Hierarchical Clustering Analyses

**Figure 3:**
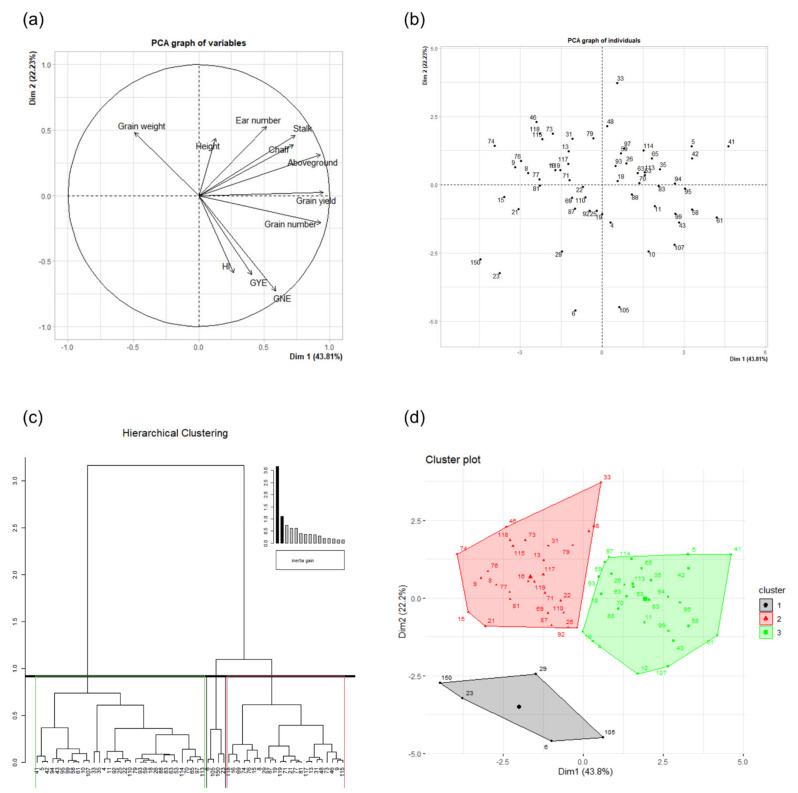
Hierarchical clustering based on principal component analysis (PCA) for the 60 wheat genotypes grown under elevated CO_2_ and high-temperature conditions. *GNE*: grain number ear^−1^; *GYE*: grain yield ear^−1^; *HI*: harvest index. (**a**) The *variables plot* represents the correlation of traits with the PCA axes. (**b**) The *individuals plot* exhibits the position of genotypes in the PCA. (**c**) *Hierarchical clustering* of genotypes based on their distribution on the PCA. (**d**) *Cluster plot* of genotypes distributed in the PCA. PCA and hierarchical clustering were performed using individuals as the mean of the four replicates (*n* = 4) for each genotype.

The natural variation in plant biomass and grain yield within the 60 wheat genotypes was further investigated using principal component analysis (PCA). Graphs of variables and individuals from PCA are shown in Figure 3a,b. Almost all the variables under study were positively correlated with the first dimension of the PCA (Table 2). Grain yield, grain number and aboveground biomass per plant were the most commonly contributing eigenvectors (18.85, 18.07 and 18.01, respectively) and correlated variables (0.95, 0.93 and 0.93) with the first dimension of the PCA. The stalk and chaff biomasses, GNE and ear number were also highly correlated variables, with correlation values ranging between 0.74 and 0.52, followed by GYE, HI and plant height. The grain weight was the only variable that was negatively correlated with the first dimension of the PCA (−0.49). Weaker positive correlations were found for the second dimension of the PCA, while the number of negatively correlated variables increased, together with their intensity. GNE, GYE and HI were the most contributing variables (21.58, 14.96 and 14.24) and the highest negatively correlated ones (−0.73, −0.61 and −0.59) with this second dimension. Grain number per plant was also negatively correlated with the second dimension (−0.21), although it barely contributed to this dimension (1.81). Ear number, grain weight and stalk biomass were the most positively correlated variables (0.53−0.46), followed by the height of the plant and the chaff and aboveground biomasses. The grain yield barely correlated with this second dimension (0.02).

**Figure 4:**
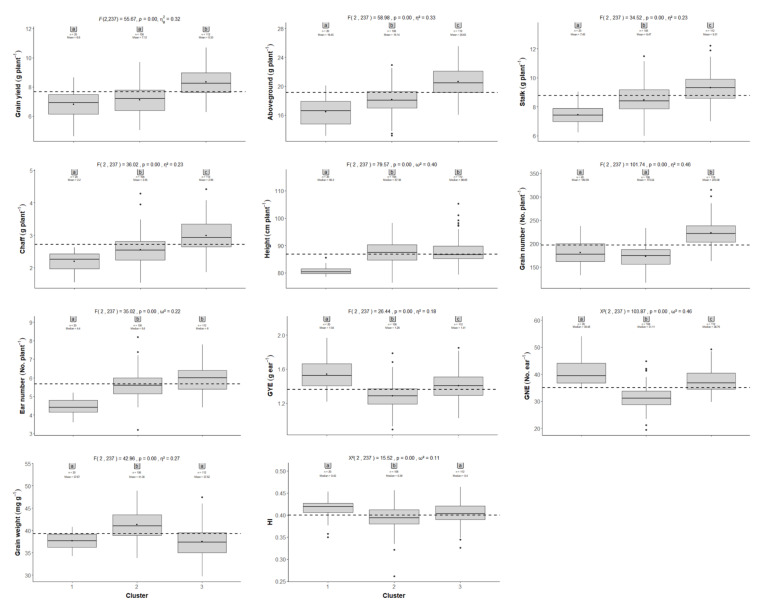
Boxplot with ANOVA of wheat production and grain yield components for the three defined clusters of the 60 wheat genotypes grown under elevated CO_2_ and high-temperature conditions. *GNE*: grain number ear^−1^; *GYE*: grain yield ear^−1^; *HI*: harvest index. Black dots represent the mean of the replicates per cluster (Cluster 1, *n* = 20; Cluster 2, *n* = 108; Cluster 3, *n* = 112). The black dotted line represents the mean for all the genotypes and replicates (*N* = 240). Statistics (*F* and *X^2^*), degrees of freedom, *p*-value (*p*) and size of the effect (η² and **ω**²) for one-way ANOVA are added. Among columns, numbers followed by the same letter are not significantly different at *p* < 0.05 for post-hoc tests.

Hierarchical clustering on principal components (HCPC) for the 60 wheat genotypes was performed, based on the PCA results. Cutting of the tree defined three major clusters (clusters 1, 2 and 3; Figure 3c). Grouped by clusters, the distribution of the genotypes in the PCA is shown in Figure 3d, whereas Figure 4 shows boxplots of wheat production and grain yield components with a one-way analysis of variance (ANOVA) for clusters. Cluster 3 included the 28 genotypes most related to grain yield, aboveground biomass and grain number, whereas the 27 genotypes from cluster 2 were mainly associated with grain weight. Cluster 1 was composed of 5 genotypes, including genotype 150 (Gazul).

The one-way ANOVA found statistically significant differences for wheat production and grain yield components when the three clusters were compared. Cluster 3 showed significantly higher grain yield and grain number per plant than clusters 2 and 1, whereas no differences between the latter two were found. Aboveground, stalk and chaff biomasses were also significantly greater for cluster 3 than for clusters 2 and 1, as well as for cluster 2 than for cluster 1. Cluster 2 showed higher grain weight than clusters 1 or 3 but lower HI, with non-significant differences between clusters 1 and 3 for any variable. Furthermore, cluster 2 also had lower GYE and GNE than clusters 1 and 3, whereas cluster 1 had the highest. Finally, cluster 1 showed the lowest ear number and plant height, with no differences between clusters 2 and 3. All the traits under study exhibited a large effect ranging between 0.18−0.57, except for HI, which showed a middle-sized effect (0.06).

### 2.5. Days from Sowing to Ear Emergence, Anthesis and Maturity

Growth duration varied among genotypes. Days from sowing until plants reached ear emergence, anthesis and maturity growth stages ranged between 57−68, 60−69 and 92−102 days after sowing, respectively (Table S2). Negative correlations were found of the average number of days until plants reached these growth stages to wheat production and grain yield components, although all of them were under |0.45| (Table S3). Weak positive correlations were found among growth durations and GYE, GNE, HI and plant height.

## 3. Discussion

Climate change will inevitably affect crop development and productivity, increasing uncertainty regarding food production. In recent years, more attention has been paid to the risks of concomitant increases in atmospheric CO_2_ concentrations and temperature for plant performance, and both factors should be assessed together to build up a realistic picture of how global warming will impact the productivity of food crops. In this context, the potential to exploit genetic variation becomes an essential strategy for the selection of crops with better adaptation to future climate conditions. So far, the effects of elevated CO_2_ when combined with increasing temperatures on wheat yield have rarely been investigated under field conditions or enclosure facilities, and most of those studies have used either a single genotype or a very limited number of cultivars [4,40,41,42,43,44,45]. Here, the genotypic variation in grain yield is investigated across a set of 60 bread wheat genotypes, using controlled environmental chambers as an approach to perform a more accurate simulation of predicted daily and seasonal variations of temperature during the wheat-growing cycle in the region of Salamanca (Spain) by the end of the century. Among the 60 wheat genotypes under study (Table 1), there were several genotypes for which the range of values in biomass production and yield-related traits were more than double the average values achieved by the lowest-ranking genotypes, showing evidence for variability in the performance of the 60 genotypes under combined elevated CO_2_ and high-temperature conditions. This implies that the selection of genotypes achieving greater productivity in a future climate scenario is feasible, as is in accordance with previous works by the authors regarding bread wheat [45], and of others regarding durum wheat [44]. It is worth noting that the CIMMYT genotypes exhibited higher biomass and grain yield when compared to Gazul (Figure 1), a result that can be interpreted as the former having better adaptability to the environment. In spite of the higher grain number per ear observed in Gazul, this was insufficient to compensate for the reduction in ear number per plant and grain weight, thus resulting in lower grain yield and biomass. Taken together, these findings suggest that the CIMMYT genotypes performed better than the Gazul genotype under combined elevated CO_2_ and high-temperature conditions, and also provide evidence for the success of breeding programs conducted under warmer temperature environments.

Likewise, we observed a strong positive correlation of grain yield with aboveground biomass and grain number (Figure 2; Table S1), as well as a highly positive correlation of grain yield with ear number and stalk and chaff biomasses; all of them, in turn, correlated with each other. These results suggest that increased grain yield was attained by a higher grain number due to a larger number of ears, but not from an increase in grain weight, as is in agreement with previous studies in wheat grown under elevated CO_2_ [18,46,47,48]. Data from the chamber experiments [46] also reported increased grain numbers per ear, but not the FACE experiments [49]. As is consistent with our finding, the grain number per square meter was the most important yield component, accounting for the effects of elevated CO_2_ and temperature in wheat and rice [4]. Altogether, these findings suggest that tillering capacity, and hence the increased number of fertile tillers, is an important factor in yield response to elevated CO_2_ [26,50], especially under higher temperatures and dryland conditions [40,44]. In a study comprising 20 wheat genotypes grown in glasshouses and controlled environment chambers, it was also found that CO_2_ enrichment stimulated tillering, regardless of the cultivars [51]. For nine durum wheat varieties grown hydroponically, Sabella et al. (2020) [44] observed a higher yield for the old cultivar, Cappelli, when compared to modern cultivars under elevated CO_2_ and high temperatures, and this was associated with a larger number of ears per plant rather than to changes within the ears. These data resemble previous studies showing a greater response to elevated CO_2_ by older wheat varieties than the more recently released ones, because of the higher plasticity of the former in tiller production [26,52]. Evaluating plant developmental responses to CO_2_ enrichment has underlined the importance of sink strength at the whole plant level, both as a mechanism to increase plant growth and to avoid photosynthetic acclimation [23]. Enhanced leaf carbohydrate content caused by higher photosynthesis rates under CO_2_ enrichment might be efficiently used for the development of further sinks, such as new tillers, to distribute the photoassimilates [53]. Consequently, this ability to develop new tillers when an extra carbon source is made available is in accordance with the previous suggestion that ensuring adequate sink strength is essential for maximizing the response to elevated CO_2_.

Furthermore, we observed that grain weight was negatively correlated with grain number per plant and per ear (Figure 2; Table S1), indicative of the change in size distribution toward smaller grains, as previously shown in wheat plants grown under elevated CO_2_ [18]. This finding is in contrast to the slight shifts toward a larger grain size, as reported by Högy et al. (2013) [54], and the increase in grain size found in three wheat cultivars by Panozzo et al. (2014) [55], although most previous results from FACE experiments showed slight changes in grain weight or that it simply remained unchanged [48,49]. Such contradictory results in wheat grain weight could be associated with the co-influence of other factors related to differences in growing conditions [18,56], such as water availability [47,57]. Increased temperature adversely impacts grain development, owing to several limitations in assimilate supply, the length of the grain-filling period, and the starch biosynthesis and deposition rates, together leading to smaller grains [32,58]. Wheat is most sensitive to abrupt heat stress around flowering, and becomes more tolerant to higher temperatures during the grain-filling stage [59]. In line with this observation, Wollenweber et al. (2003) [60] found distinct effects on grain weight according to the timing of heat application. Weichert et al. (2017) [61] reported the adverse impact of a post-anthesis heatwave on wheat grain yield, being responsible for reduced grain size in the last term. In addition, it is worthy of highlighting that central spikelets and proximal florets flower earlier and receive priority in the deposition of assimilates, and thus tend to have a larger grain size when compared to distal spikelets and florets [58,62]. Higher temperatures may induce upper and lower spikelets and distal florets to abort or develop constitutively smaller grains; this may, therefore, bring together a greater heterogeneity in the crop grain size and a shift in the distribution toward smaller grain size [58]. Even genetic differences among cultivars may contribute to the inconsistent results on wheat grain under elevated CO_2_ [50]. Among wheat genotypes, a negative relationship between grains per square meter and grain weight has frequently been observed [63]. In fact, with the advent of the semidwarf cultivars, there was a trend toward a decrease in grain weight, accompanied by more grains in the distal positions in spikelets having a lower potential grain weight [64]. These observations may explain the negative relationship between grain weight and yield components, including the grain number per plant and per ear, as observed in our study, which is not related to competition among grains for assimilates but to the increasing contribution of grains of low potential weight that are, thereby, smaller [65,66].

From the hierarchical clustering analysis (Figure 3c), we inferred that the wheat genotypes were grouped into three clusters with similar variation sources in each of them (Figure 3d), although differences in biomass and yield-related traits among clusters were identified in line with the PCA analysis (Figure 3a,b). These findings suggest that there were contrasting yield strategies among the wheat genotypes under the studied environmental conditions. Despite the larger number of grains per ear exhibited by genotypes of cluster 1, and the improved grain weight of those of cluster 2, the highest yielding genotypes were found within cluster 3, in association with the highest aboveground, stalk and chaff biomasses, grain number, and ears per plant (Figure 4). This suggests that genotypes that can preferentially increase these yield-related traits will lead to better yield response under combined elevated CO_2_ and high temperature. In line with this observation, most of the previous studies worldwide have shown that grain yield progress in wheat was mostly correlated with grain number per square meter rather than grain weight [67,68,69]. Similarly, in Spain, the bread wheat genetic improvement on yield from 1930 to 2000 was also achieved by an increase in grain number, while grain weight was unchanged [70]. In fact, the introduction of semidwarf cultivars throughout the 1960s increased grains per square meter by increasing assimilate allocation to the ear during the pre-flowering period, allowing more floret primordia to become fertile florets [71]. Therefore, past genetic gains in bread wheat yield have been widely linked to an increase in harvest index and a decline in plant height [72]. Overall, improved semidwarf high-yielding bread wheat varieties from Mexico and European countries, mostly France and Italy, have played a major role in yield improvements in Spain [73], where the scarcity of rain is frequent in spring when temperatures rise rapidly. Thus, the incorporation of drought tolerance has always been one of the main goals of Spanish wheat-breeding programs, because drought and high temperature are the prevalent stresses during the reproductive stages constraining wheat production in Spain [74]. During the 1990s, the private seed sector successfully introduced and marketed 74 bread wheat varieties, including Gazul, which is currently cultivated in Spain [73]. Tillering is regulated genotypically, but it is also influenced by the environment [75]. In this context, tillering reduction has been proposed as a suitable feature under terminal drought stress because it decreases soil water use before anthesis [76]. Improved tiller economy could enhance the partitioning of assimilates to the ear, leading to increased grain yield per ear, mainly resulting from higher grain number per ear, but decreased grain per square meter [76,77]. In accordance with these findings, cluster 1 grouped the lowest-yielding genotypes, driven by a decreased number of productive tillers with improved ear fertility but decreased grain yield. The fact that genotype 150 (Gazul) was closely related to four CIMMYT genotypes highlights the important contribution of CIMMYT germplasm in the release of Spanish varieties during the last century [70,73], as reported by the seed company (Limagrain Iberica SA) involved in the marketing of improved bread varieties. It is interesting, however, that the genotypes grouped into cluster 2 seem to sustain grain yield production by higher grain weight, to compensate for reduced grain numbers per ear. Therefore, these intermediate yielding genotypes, when compared with the lowest and highest yielding genotypes, had different attributes with heavier grains rather than higher grain numbers per square meter. In agreement with these results, it has been found that the grain yield progress of CIMMYT advanced lines from 1977 to 2008 was driven by higher grain weight, rather than more grains per square meter [78]. Other earlier studies have also reported that grain weight has contributed to yield progress [79,80].

Breeding programs have focused on developing germplasm adapted to different crop-producing geographic regions and, in Mediterranean areas, the earliness of heading has been recognized as an adaptive trait of modern cultivars aiming to escape drought/heat terminal stress [77,81]. Indeed, Lopes et al. (2012) [78] reported that, in warmer environments, the reduction in days until heading displayed in high-yielding spring wheat advanced lines allowed an escape from drought/heat episodes at the most sensitive stage of grain setting. In our study, there were differences among genotypes in the number of days from sowing to ear emergence, which could partly explain the better performance under the studied conditions of the early-heading genotypes when compared to the late-heading ones (Table S2). Nevertheless, genotype 150 (Gazul) performed poorly under combined elevated CO_2_ and high temperature, although it was one of the earliest-heading genotypes. Hence, other features should be considered, because phenology does not appear to be the only cause of variability in grain yield (Table S3). It must be highlighted that one of the highest yielding genotypes in our study (genotype 5) has also exhibited good and stable performance under warm temperatures in previous studies [82].

## 4. Materials and Methods

### 4.1. Plant Material and Experimental Conditions

The experiment was carried out with 60 bread wheat genotypes (*Triticum aestivum* L.). Fifty-nine genotypes were selected to represent the germplasm of the CIMMYT heat-tolerance wheat screening nursery (8TH HTWSN), together with the Gazul genotype (referred to as genotype 150), with a high yield and adaptability to the Mediterranean climate of the Salamanca region [10,83,84], as well as being identified as a genetic source of excellent bread-making quality owing to the high protein content and specific glutenin and gliadin quality of their grain [73] (Table S4). The genotypes of the 8TH HTWSN were previously selected for high performance in warmer temperatures [85]. Seeds were sown in 5-L pots, with a density of five plants per pot after emergence. Pots were filled with 1.2 kg of a mixture of peat:perlite (4:1), and 4 g of both KNO_3_ and KH_2_PO_4_ were added to each pot, with the peat providing enough provision of other nutrients [86]. Pots were placed in two, 3.6-m length × 4.8-m width × 2.4-m height, controlled environment chambers maintained on a 16-/8-hour light/dark regime with an irradiance of 400 µmol m^−2^ s^−1^ at the top of the canopy, provided by a combination of blue- plus red-peak fluorescent lamps, and relative humidity of 40%/60% day/night. The atmospheric CO_2_ concentration was set at 700 µmol mol^−1^ by injecting pure CO_2_ [87,88]. The temperature was 4 °C above current temperatures, simulating the daily and seasonal oscillations of typical temperatures in the natural environments of the Salamanca region [45]. Four different sections were established to reproduce the daily temperature oscillations: night, and the initial, central and final parts of the photoperiod. These temperatures were increased by three levels reproducing the natural seasonal oscillations throughout wheat development, when at least 50% of the plants reached ear emergence and anthesis, respectively. The experiment was conducted in a completely randomized design, with four replicates/pot per each of the studied genotypes. Once germinated, 1200 plants were grown in 240 pots. Water was supplied during crop development three times per week to maintain pot field capacity, and the pots were rotated twice a week in order to avoid edge effects.

### 4.2. Evaluation of Phenological Traits

In the 60 bread wheat genotypes, through periodic observation (three times per week) the following traits were evaluated: the number of days from sowing to ear emergence (days until heading) was recorded when 50% of the plants per each of the studied genotypes had fully emerged ears; days from sowing to anthesis were determined when 50% of ears showed extruded anthers along the head; the days from anthesis to maturity were also evaluated. After anthesis, plant height was determined as the distance from the ground to the ear tip, excluding awns, of the main stem of two plants per pot per each of the studied genotypes.

### 4.3. Harvesting and Yield Component Determination

At physiological maturity, the aboveground plant parts were harvested from each pot and divided into stalks (stems and leaves) and ears. Grains and chaff components were separated from the ears by manual threshing. The number of ears and grains per plant and per ear were determined, and the dry weights for the stalk, chaff, and the grain yield per plant and per ear were recorded after drying in an oven at 60 °C over 48 h. The grain weight was estimated as the quotient between the grain yield and the grain number per plant. The harvest index (HI) was also determined as the ratio of grain yield to total aboveground biomass.

### 4.4. Statistical Analysis

The normal distribution of all parameters under study was tested using the Shapiro–Wilk test from the function *tapply* of the *base* package in the statistical software *R* [89]. Descriptive statistics of variables were measured using the function *describBy* from the *psych* package [90]. A multiple analysis of variance (MANOVA) was performed to determine differences for each studied trait among the 60 genotypes employed, and their four replicates per genotype (*N* = 240), using the function *manova* of the package *stats* [89]. A one-sample test was carried out for comparison of the wheat production and grain yield components between the average values of the Gazul genotype (*n* = 4) and the 59 wheat genotype population belonging to CIMMYT (*n* = 236). A PCA was applied to the genotype by a trait (GxT) matrix of means, as described in Driever et al. (2014) [5]. The principal components performed were employed as a pre-process for genotype clustering (hierarchical clustering on principal components (HCPC)) as suggested by Husson et al. (2010) [91]. Clusters grouped a different number of genotypes, with four replicates per genotype (Cluster 1: 5 genotypes, *n* = 20; Cluster 2: 27 genotypes, *n* = 108; Cluster 3: 28 genotypes, *n* = 112). The test of Levene for homogeneity of variance was conducted to determine homoscedasticity among clusters in the traits under study, using the package *DescTools* [92]. For comparations of the traits among clusters, a one-way ANOVA for independent measures was performed. Table S5 schematizes decisions taken to the choice of the best one-sample or one-way ANOVA test, together with the interpretation for the values of the effect size. Correlation networks were performed with the packages *psych* and *reshape2* [91,93], along with the software “Cytoscape” [94], using a threshold for the Spearman’s correlation values of r ≥ |0.45|. Correlation coefficients for wheat production and grain yield components were also calculated, using the 60 genotypes employed and their replicates (*N* = 240), whereas, for correlations of these traits with the days to heading, only the mean values for each genotype were employed. For the whole study, differences were considered significant at *p* < 0.05.

## 5. Conclusions

Crops are predicted to be exposed more frequently to high temperatures in the future as a consequence of climate change. Therefore, the exploration of variability in wheat productivity under combined elevated CO_2_ and high temperatures is essential for the selection of genotypes that are better adapted to future global warming. The dissection of yield-related traits in our study discloses that the bread wheat genotypes employed diverse strategies to achieve final grain yield under elevated CO_2_ levels combined with a high temperature, most probably as a consequence of the contrasting breeding strategies used to improve grain yield throughout the 20th century. Increased grain yield was driven by grain numbers and ears per plant, rather than per grain weight. The CIMMYT genotypes performed better than the Gazul genotype under combined elevated CO_2_ and high temperature, providing evidence for the success of the breeding programs under warmer temperature environments. Further research is needed to investigate whether the highest yielding genotypes identified in the present study will be able to maintain their yield advantage, regardless of nutrient availability or drought stress, combined with high temperature, for the selection of widely adapted climate-resilient wheat genotypes.

## Figures and Tables

**Table 1:** Wheat production (aboveground, stalk and chaff biomasses), grain yield components (grain yield, grain number, ear number, grain weight, grain yield per ear, grain number per ear and harvest index), and plant height of sixty wheat genotypes grown under elevated CO_2_ and high-temperature conditions.

Genotype	Grain Yield (g plant^−1^)			Above-Ground (g plant^−1^)			Stalk (g plant^−1^)			Chaff (g plant^−1^)			Height (cm plant^−1^)			Grain Number (No. plant^−1^)			Ear Number (No. plant^−1^)			Grain Weight (mg plant^−1^)			GYE (g ear^−1^)			GNE (No. ear^−1^)			HI		
150	5.45	±	1.05	14.38	±	1.64	6.82	±	0.44	2.12	±	0.35	80.36	±	0.77	156.10	±	32.80	4.10	±	0.48	34.97	±	0.81	1.32	±	0.10	37.79	±	3.34	0.38	±	0.03
74	6.18	±	0.68	16.29	±	1.82	8.23	±	1.26	1.88	±	0.30	89.71	±	1.21	151.79	±	19.87	5.65	±	0.30	40.95	±	3.61	1.10	±	0.12	26.98	±	4.23	0.38	±	0.04
23	6.42	±	0.53	15.17	±	1.31	6.83	±	0.48	1.91	±	0.35	79.86	±	0.19	159.37	±	12.57	4.45	±	0.57	40.27	±	0.42	1.45	±	0.08	36.02	±	2.24	0.42	±	0.01
8	6.46	±	1.22	17.14	±	3.79	7.88	±	1.72	2.80	±	0.86	81.49	±	3.04	161.55	±	32.03	5.30	±	1.16	40.04	±	1.60	1.22	±	0.06	30.58	±	1.55	0.38	±	0.01
76	6.50	±	0.77	17.12	±	1.72	8.46	±	0.86	2.16	±	0.26	87.15	±	2.16	160.50	±	24.84	5.40	±	0.63	40.63	±	1.60	1.20	±	0.07	29.70	±	2.47	0.38	±	0.02
15	6.53	±	0.85	16.11	±	2.41	7.18	±	1.27	2.39	±	0.39	77.14	±	1.38	158.58	±	22.88	5.55	±	0.53	41.28	±	1.08	1.18	±	0.09	28.51	±	2.19	0.41	±	0.02
73	6.59	±	0.94	18.81	±	1.49	9.59	±	0.63	2.63	±	0.23	91.38	±	0.59	158.99	±	15.40	4.95	±	0.10	41.34	±	2.39	1.33	±	0.18	32.14	±	3.25	0.35	±	0.03
21	6.62	±	0.59	16.35	±	1.07	7.78	±	0.28	1.94	±	0.27	88.73	±	1.92	163.79	±	8.89	5.00	±	0.52	40.44	±	2.71	1.34	±	0.19	32.94	±	2.81	0.40	±	0.01
9	6.64	±	0.73	17.19	±	1.80	8.18	±	0.66	2.38	±	0.44	88.13	±	3.34	148.69	±	18.14	4.95	±	0.55	44.71	±	1.92	1.34	±	0.05	30.04	±	1.74	0.39	±	0.01
118	6.67	±	0.23	17.19	±	1.40	8.00	±	0.95	2.53	±	0.43	84.70	±	4.05	176.82	±	4.29	6.80	±	0.49	37.75	±	1.48	0.99	±	0.08	26.07	±	1.32	0.39	±	0.03
81	6.75	±	1.03	17.15	±	2.14	8.01	±	1.27	2.39	±	0.07	94.69	±	2.02	164.49	±	28.73	5.00	±	1.40	41.20	±	2.23	1.40	±	0.26	33.88	±	5.17	0.39	±	0.02
77	6.86	±	0.96	17.42	±	1.88	8.49	±	0.63	2.07	±	0.34	91.03	±	4.86	168.32	±	19.71	5.25	±	0.64	40.66	±	1.95	1.31	±	0.19	32.32	±	4.74	0.39	±	0.02
115	6.92	±	0.94	18.23	±	1.64	8.64	±	0.84	2.67	±	0.25	87.08	±	2.59	160.01	±	25.82	5.55	±	0.44	43.49	±	2.81	1.25	±	0.10	28.80	±	3.72	0.38	±	0.03
87	6.96	±	1.43	18.14	±	3.64	8.62	±	1.82	2.55	±	0.48	87.36	±	1.30	184.34	±	43.52	4.90	±	0.48	38.03	±	2.24	1.44	±	0.35	38.03	±	9.69	0.38	±	0.01
29	7.04	±	0.77	17.26	±	1.00	7.89	±	0.21	2.33	±	0.07	82.15	±	2.20	184.91	±	16.88	4.75	±	0.34	38.03	±	1.02	1.48	±	0.08	38.90	±	1.70	0.41	±	0.02
46	7.06	±	0.32	18.39	±	0.84	8.23	±	0.57	3.11	±	0.27	88.99	±	3.74	147.54	±	7.81	5.45	±	0.10	47.84	±	1.22	1.30	±	0.07	27.09	±	1.81	0.38	±	0.02
119	7.09	±	0.90	18.33	±	1.63	8.84	±	0.86	2.40	±	0.23	92.60	±	1.93	170.30	±	5.50	5.10	±	0.35	41.63	±	5.21	1.39	±	0.15	33.50	±	2.44	0.39	±	0.02
6	7.20	±	0.75	16.94	±	1.73	7.51	±	0.75	2.23	±	0.33	79.49	±	1.19	194.79	±	17.84	4.35	±	0.34	36.91	±	0.71	1.65	±	0.08	44.78	±	2.35	0.42	±	0.01
110	7.25	±	0.81	18.25	±	1.07	8.77	±	0.43	2.24	±	0.06	85.64	±	3.54	202.72	±	19.43	5.75	±	0.38	35.73	±	1.16	1.26	±	0.07	35.21	±	1.56	0.40	±	0.02
71	7.29	±	0.65	18.70	±	0.87	9.02	±	0.57	2.39	±	0.34	93.69	±	1.18	169.53	±	12.76	4.85	±	0.34	42.97	±	1.26	1.50	±	0.10	35.03	±	2.76	0.39	±	0.03
16	7.34	±	0.32	17.69	±	0.36	7.94	±	0.12	2.40	±	0.20	83.21	±	3.08	181.28	±	9.93	6.35	±	0.38	40.54	±	1.26	1.16	±	0.08	28.58	±	1.32	0.42	±	0.01
22	7.44	±	0.79	18.10	±	1.96	8.10	±	1.11	2.56	±	0.26	85.10	±	0.27	199.14	±	21.75	6.15	±	0.60	37.39	±	0.74	1.21	±	0.07	32.38	±	1.56	0.41	±	0.02
13	7.49	±	1.01	18.87	±	2.29	8.43	±	1.13	2.95	±	0.32	89.96	±	0.86	166.74	±	23.43	5.45	±	0.68	45.03	±	3.37	1.38	±	0.15	30.67	±	3.44	0.40	±	0.02
92	7.50	±	0.68	18.40	±	1.24	8.47	±	0.43	2.43	±	0.27	83.39	±	1.90	199.36	±	17.06	5.60	±	0.37	37.61	±	0.98	1.34	±	0.11	35.69	±	3.41	0.41	±	0.01
117	7.53	±	0.35	18.56	±	1.17	8.42	±	0.58	2.60	±	0.34	89.48	±	0.78	173.98	±	5.18	5.65	±	0.19	43.31	±	1.77	1.33	±	0.07	30.80	±	0.52	0.41	±	0.01
79	7.56	±	0.91	19.75	±	2.48	9.12	±	1.34	3.06	±	0.31	84.79	±	2.56	181.24	±	19.11	5.95	±	0.41	41.69	±	1.43	1.27	±	0.14	30.47	±	2.52	0.38	±	0.02
33	7.60	±	1.35	21.98	±	0.67	10.76	±	0.66	3.61	±	0.47	85.28	±	4.74	168.38	±	37.34	5.65	±	0.41	45.58	±	3.37	1.36	±	0.30	30.02	±	7.23	0.35	±	0.06
69	7.66	±	1.50	17.81	±	2.54	7.63	±	0.89	2.51	±	0.45	79.86	±	2.44	193.14	±	28.90	6.35	±	0.85	39.48	±	1.79	1.21	±	0.20	30.62	±	4.64	0.43	±	0.03
31	7.71	±	1.26	18.71	±	2.69	8.27	±	1.09	2.73	±	0.36	87.35	±	3.87	177.91	±	29.82	6.50	±	1.16	43.38	±	2.12	1.20	±	0.17	27.52	±	3.13	0.41	±	0.01
19	7.73	±	0.66	19.04	±	1.21	8.74	±	0.58	2.57	±	0.10	87.91	±	3.11	193.25	±	3.79	5.10	±	0.38	40.03	±	3.77	1.52	±	0.11	38.04	±	2.65	0.41	±	0.01
114	7.75	±	0.32	20.64	±	1.03	10.42	±	0.53	2.46	±	0.37	100.68	±	3.35	214.96	±	15.27	5.65	±	0.34	36.11	±	1.47	1.37	±	0.04	38.03	±	0.75	0.38	±	0.01
97	7.79	±	0.35	19.89	±	1.06	9.42	±	0.50	2.68	±	0.35	88.10	±	3.37	213.71	±	8.05	6.55	±	0.19	36.49	±	2.51	1.19	±	0.06	32.67	±	2.01	0.39	±	0.01
113	7.79	±	0.87	20.01	±	2.47	9.91	±	1.27	2.31	±	0.39	90.13	±	4.00	237.19	±	44.64	6.40	±	1.02	33.16	±	2.30	1.22	±	0.06	36.96	±	1.32	0.39	±	0.01
25	7.81	±	0.47	18.72	±	1.04	8.22	±	0.64	2.69	±	0.16	83.50	±	4.10	197.07	±	12.53	5.60	±	0.28	39.63	±	0.68	1.40	±	0.10	35.23	±	2.36	0.42	±	0.02
18	7.83	±	1.28	19.61	±	2.49	8.82	±	1.01	2.97	±	0.61	87.00	±	2.85	202.67	±	31.10	5.75	±	0.98	38.62	±	2.16	1.37	±	0.13	35.37	±	2.38	0.40	±	0.04
70	7.84	±	1.17	19.75	±	3.23	8.98	±	1.72	2.93	±	0.73	93.44	±	3.93	220.48	±	43.49	5.85	±	1.23	35.89	±	3.65	1.37	±	0.24	37.98	±	4.14	0.40	±	0.04
63	7.89	±	0.82	20.21	±	1.54	9.23	±	0.77	3.09	±	0.31	85.29	±	2.36	215.34	±	14.60	6.00	±	0.43	36.59	±	1.61	1.32	±	0.10	35.96	±	2.46	0.39	±	0.03
105	7.91	±	0.89	18.50	±	1.33	8.21	±	0.60	2.38	±	0.12	82.40	±	2.10	207.79	±	28.23	4.40	±	0.00	38.17	±	1.69	1.80	±	0.20	47.22	±	6.42	0.43	±	0.02
88	8.00	±	0.78	20.27	±	1.31	9.21	±	0.79	3.05	±	0.23	87.35	±	2.97	200.75	±	19.37	5.25	±	0.34	39.88	±	1.76	1.53	±	0.22	38.35	±	4.41	0.39	±	0.02
93	8.01	±	1.51	20.01	±	3.21	9.01	±	1.47	2.99	±	0.53	88.54	±	1.84	192.90	±	23.42	5.70	±	0.60	41.28	±	3.34	1.40	±	0.17	33.84	±	1.97	0.40	±	0.03
35	8.02	±	1.11	21.93	±	2.59	10.30	±	1.39	3.61	±	0.59	87.33	±	1.71	195.70	±	8.82	4.90	±	0.62	40.91	±	4.55	1.63	±	0.02	40.28	±	3.92	0.37	±	0.03
4	8.06	±	0.63	18.18	±	0.90	7.45	±	0.38	2.67	±	0.05	85.81	±	1.84	219.29	±	9.45	6.25	±	0.19	36.71	±	1.34	1.29	±	0.08	35.09	±	1.15	0.44	±	0.02
26	8.12	±	0.44	20.35	±	1.49	9.58	±	0.91	2.65	±	0.24	87.34	±	1.58	204.17	±	6.84	6.05	±	0.41	39.77	±	0.95	1.34	±	0.03	33.81	±	1.15	0.40	±	0.01
48	8.15	±	0.43	20.28	±	1.18	9.34	±	0.76	2.79	±	0.28	90.09	±	4.08	185.85	±	20.06	6.35	±	0.47	44.08	±	2.62	1.29	±	0.10	29.31	±	2.95	0.40	±	0.01
99	8.19	±	0.63	20.80	±	1.14	9.62	±	0.23	3.00	±	0.52	88.19	±	1.78	238.56	±	11.01	5.50	±	0.26	34.34	±	2.81	1.49	±	0.10	43.39	±	1.40	0.39	±	0.02
53	8.19	±	0.72	20.25	±	1.45	8.78	±	0.76	3.27	±	0.33	82.55	±	1.55	221.46	±	14.92	6.40	±	0.33	36.96	±	0.85	1.28	±	0.12	34.65	±	2.60	0.40	±	0.02
59	8.24	±	1.32	20.47	±	2.57	9.20	±	1.02	3.03	±	0.33	86.99	±	0.61	192.70	±	29.26	5.95	±	0.70	42.73	±	1.07	1.38	±	0.10	32.33	±	1.78	0.40	±	0.01
83	8.28	±	0.56	21.10	±	2.28	9.70	±	1.19	3.12	±	0.58	85.04	±	7.27	216.33	±	16.38	5.70	±	0.84	38.31	±	0.37	1.47	±	0.23	38.52	±	6.17	0.39	±	0.02
10	8.31	±	0.98	19.66	±	2.19	8.43	±	0.97	2.92	±	0.33	82.31	±	2.65	226.90	±	26.78	5.40	±	0.78	36.71	±	2.78	1.55	±	0.15	42.14	±	1.38	0.42	±	0.02
65	8.35	±	0.89	20.65	±	2.87	9.13	±	1.67	3.17	±	0.54	91.61	±	5.93	217.74	±	8.02	6.30	±	0.62	38.29	±	2.87	1.33	±	0.16	34.81	±	3.61	0.41	±	0.03
42	8.51	±	0.65	21.34	±	1.32	9.34	±	0.87	3.49	±	0.22	88.88	±	0.71	250.79	±	12.25	6.80	±	0.52	33.91	±	1.12	1.25	±	0.09	36.96	±	1.86	0.40	±	0.02
107	8.56	±	1.37	20.33	±	3.43	9.10	±	1.42	2.68	±	0.74	84.05	±	2.79	249.29	±	47.63	5.75	±	0.50	34.48	±	1.63	1.49	±	0.16	43.14	±	5.03	0.42	±	0.01
11	8.65	±	0.21	20.15	±	0.90	8.78	±	0.72	2.72	±	0.21	86.24	±	2.95	232.13	±	13.49	6.30	±	0.62	37.33	±	1.50	1.38	±	0.11	36.96	±	1.64	0.43	±	0.02
58	8.70	±	0.81	21.47	±	2.01	9.85	±	0.82	2.92	±	0.56	86.29	±	1.27	247.08	±	13.25	5.90	±	0.50	35.16	±	1.55	1.48	±	0.08	41.99	±	2.19	0.41	±	0.02
5	8.76	±	1.17	22.09	±	2.40	9.88	±	0.86	3.46	±	0.40	84.75	±	1.54	238.93	±	21.61	6.80	±	0.59	36.60	±	3.13	1.28	±	0.09	35.13	±	0.78	0.40	±	0.01
43	8.97	±	0.99	20.86	±	1.66	8.64	±	0.77	3.26	±	0.13	89.44	±	1.97	232.37	±	31.09	5.65	±	0.25	38.77	±	3.25	1.58	±	0.12	41.12	±	5.22	0.43	±	0.02
94	8.99	±	0.68	21.36	±	1.95	9.59	±	1.18	2.79	±	0.28	94.06	±	3.87	226.96	±	24.88	6.00	±	0.59	39.71	±	1.44	1.50	±	0.12	37.88	±	3.00	0.42	±	0.02
95	9.00	±	0.66	21.73	±	2.01	9.59	±	1.04	3.14	±	0.44	85.65	±	3.43	231.89	±	18.72	6.15	±	1.00	38.84	±	1.48	1.48	±	0.15	38.15	±	3.88	0.41	±	0.01
61	9.03	±	0.71	22.21	±	1.52	10.27	±	1.23	2.92	±	0.72	85.68	±	1.24	259.49	±	13.43	5.90	±	0.62	34.75	±	1.25	1.53	±	0.09	44.23	±	3.49	0.41	±	0.01
41	9.68	±	0.85	23.25	±	1.59	9.80	±	0.73	3.78	±	0.22	82.73	±	3.39	252.73	±	27.81	7.25	±	0.44	38.38	±	1.53	1.33	±	0.05	34.82	±	2.47	0.42	±	0.01
Mean	7.66	±	1.12	19.16	±	2.51	8.78	±	1.20	2.72	±	0.55	86.82	±	4.88	197.01	±	36.05	5.67	±	0.84	39.26	±	3.68	1.36	±	0.19	34.99	±	5.63	0.40	±	0.03
Min	4.63			13.13			5.99			1.54		76.35				116.39		3.20				29.73	0.89			19.40				0.26			
Max	10.70			25.50			12.21			4.42		105.30				315.33		8.20				48.93	1.96			54.02				0.46			
Q1	6.96			17.58			7.95			2.36		83.80				171.31		5.00				36.87	1.24			31.23				0.39			
Q3	8.43			21.03			9.65			3.04		89.75				221.81		6.20				41.23	1.48			38.22				0.42			
Skewness	−0.02			0.02			0.14			0.37		0.41				0.27		0.09				0.36	0.29			0.32				−0.7			
Kurtosis	−0.25			−0.23			−0.28			−0.08		0.51				−0.10		−0.06				−0.10	0.27			0.27				2.68			
*p*-value	4.08 × 10^−11^			8.82 × 10^−12^			6.67 × 10^−10^			8.18 × 10^−17^		6.38 × 10^−30^				2.85 × 10^−25^		1.37 × 10^−16^				2.85 × 10^−28^	4.72 × 10^−14^			2.32 × 10^−28^				5.22 × 10^−6^			

GNE: grain number ear-1; GYE: grain yield ear-1; HI: harvest index; Max: maximum; Min: minimum; Q1 and Q3: first and third quartile. For each genotype, values represent the mean ± standard deviation (SD) of four replicates (n = 4). Genotypes are ranked according to the increasing mean of grain yield. Within columns, all genotypes and replicates (N = 240) were employed for the calculation of the total mean ± SD (Mean), Skewness and Kurtosis. Statistical significance (*p*-value) was calculated by multivariate analysis of variance (MANOVA). 
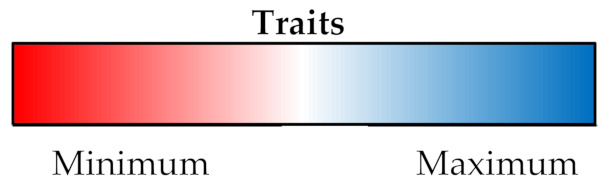

**Table 2:** Correlations and contributions between the original variables with the first two dimensions of the principal component analysis.

Dim. 1				Dim. 2			
Continuous Variables	Corr.	Cos^2^	Contr.	Continuous Variables	Corr.	Cos^2^	Contr.
Grain yield	0.95	0.91	18.85	Ear number	0.53	0.28	11.39
Grain number	0.93	0.87	18.07	Grain weight	0.48	0.23	9.44
Aboveground	0.93	0.87	18.01	Stalk	0.46	0.21	8.69
Stalk	0.74	0.55	11.33	Height	0.44	0.19	7.80
Chaff	0.72	0.52	10.88	Chaff	0.39	0.15	6.09
GNE	0.59	0.35	7.18	Aboveground	0.31	0.10	4.00
Ear number	0.52	0.27	5.52	Grain yield	0.02	0.00	0.02
GYE	0.40	0.16	3.39	Grain number	−0.21	0.04	1.81
HI	0.27	0.07	1.48	HI	−0.59	0.35	14.24
Height	0.13	0.02	0.33	GYE	−0.61	0.37	14.96
Grain weight	−0.49	0.24	4.96	GNE	−0.73	0.53	21.58

*Dim.*: dimension; *Contr.*: contribution; *Corr*.: correlation; *GNE*: grain number ear^−1^; *GYE*: grain yield ear^−1^; *HI*: harvest index; *Corr.* indicates the correlation between each variable and the dimension. The squared correlation (*Cos^2^*) values between the variables and the dimensions are used to estimate the quality of the representation. *Cont.* expresses the contributions, in percentages, of each variable when accounting for the variability in the dimension. 
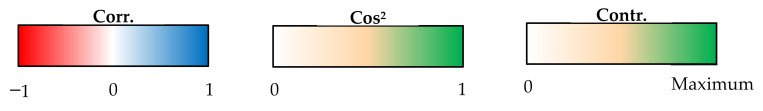

## Data Availability

Data are available upon request from the corresponding authors.

## Supplementary Materials

The following are available online at https://www.mdpi.com/article/10.3390/plants10081596/s1, Table S1: Correlation coefficient matrix for wheat production and grain yield components. Table S2: Average days from sowing to ear emergence, anthesis and maturity developmental stages. Table S3: Correlation coefficient matrix for average wheat production and grain yield components, with days from sowing to ear emergence, anthesis and maturity developmental stages. Table S4: Catalogue of the 60 wheat genotypes employed in the present study. Table S5: Statistical design with tests employed.

## References

[1] (2014). IPCC Climate Change 2014: Synthesis Report. Contribution of Working Groups I, II and III to the Fifth Assessment Report of the Intergovernmental Panel on Climate Change.

[2] NOAA-ESRL Trends in atmospheric carbon dioxide. https://www.esrl.noaa.gov/gmd/ccgg/trends/index.html.

[3] Lobell D.B., Schlenker W., Costa-Roberts J. (2011). Climate trends and global crop production since 1980. Science.

[4] Cai C., Yin X., He S., Jiang W., Si C., Struik P.C., Luo W., Li G., Xie Y., Xiong Y. (2016). Responses of wheat and rice to factorial combinations of ambient and elevated CO_2_ and temperature in FACE experiments. Glob. Chang. Biol..

[5] Driever S.M., Lawson T., Andralojc P.J., Raines C.A., Parry M.A.J. (2014). Natural variation in photosynthetic capacity, growth, and yield in 64 field-grown wheat genotypes. J. Exp. Bot..

[6] Abdelrahman M., Burritt D.J., Gupta A., Tsujimoto H., Tran L.-S.P. (2020). Heat stress effects on source–sink relationships and metabolome dynamics in wheat. J. Exp. Bot..

[7] Long S.P., Ainsworth E.A., Rogers A., Ort D.R. (2004). Rising atmospheric carbon dioxide: Plants FACE the future. Annu. Rev. Plant Biol..

[8] Ainsworth E.A., Rogers A. (2007). The response of photosynthesis and stomatal conductance to rising [CO_2_]: Mechanisms and environmental interactions. Plant Cell Environ..

[9] Pérez P., Morcuende R., Martín del Molino I., Martínez-Carrasco R. (2005). Diurnal changes of Rubisco in response to elevated CO_2_, temperature and nitrogen in wheat grown under temperature gradient tunnels. Environ. Exp. Bot..

[10] Gutiérrez D., Gutiérrez E., Pérez P., Morcuende R., Verdejo A.L., Martinez-Carrasco R. (2009). Acclimation to future atmospheric CO_2_ levels increases photochemical efficiency and mitigates photochemistry inhibition by warm temperatures in wheat under field chambers. Physiol. Plant..

[11] Pérez P., Alonso A., Zita G., Morcuende R., Martínez-Carrasco R. (2011). Down-regulation of Rubisco activity under combined increases of CO_2_ and temperature minimized by changes in Rubisco kcat in wheat. Plant Growth Regul..

[12] Vicente R., Pérez P., Martínez-Carrasco R., Usadel B., Kostadinova S., Morcuende R. (2015). Quantitative RT-PCR platform to measure transcript levels of C and N metabolism-related genes in durum wheat: Transcript profiles in elevated [CO_2_] and high temperature at different levels of n supply. Plant Cell Physiol..

[13] Martínez-Carrasco R., Pérez P., Morcuende R. (2005). Interactive effects of elevated CO_2_, temperature and nitrogen on photosynthesis of wheat grown under temperature gradient tunnels. Environ. Exp. Bot..

[14] Aranjuelo I., Cabrera-Bosquet L., Morcuende R., Avice J.C., Nogués S., Araus J.L., Martínez-Carrasco R., Pérez P. (2011). Does ear C sink strength contribute to overcoming photosynthetic acclimation of wheat plants exposed to elevated CO_2_?. J. Exp. Bot..

[15] Taub D.R., Wang X. (2008). Why are nitrogen concentrations in plant tissues lower under elevated CO_2_?. A critical examination of the hypotheses. J. Integr. Plant Biol..

[16] Carlisle E., Myers S., Raboy V., Bloom A. (2012). The effects of inorganic nitrogen form and CO_2_ concentration on wheat yield and nutrient accumulation and distribution. Front. Plant Sci..

[17] Leakey A.D.B., Ainsworth E.A., Bernacchi C.J., Rogers A., Long S.P., Ort D.R. (2009). Elevated CO_2_ effects on plant carbon, nitrogen, and water relations: Six important lessons from FACE. J. Exp. Bot..

[18] Högy P., Wieser H., Köhler P., Schwadorf K., Breuer J., Franzaring J., Muntifering R., Fangmeier A. (2009). Effects of elevated CO_2_ on grain yield and quality of wheat: Results from a 3-year free-air CO_2_ enrichment experiment. Plant Biol..

[19] Erbs M., Manderscheid R., Jansen G., Seddig S., Pacholski A., Weigel H.J. (2010). Effects of free-air CO_2_ enrichment and nitrogen supply on grain quality parameters and elemental composition of wheat and barley grown in a crop rotation. Agric. Ecosyst. Environ..

[20] ARP W.J. (1991). Effects of source-sink relations on photosynthetic acclimation to elevated CO_2_. Plant. Cell Environ..

[21] Del Pozo A., Pérez P., Morcuende R., Alonso A., Martínez-Carrasco R. (2005). Acclimatory responses of stomatal conductance and photosynthesis to elevated CO_2_ and temperature in wheat crops grown at varying levels of N supply in a Mediterranean environment. Plant Sci..

[22] Del Pozo A., Pérez P., Gutiérrez D., Alonso A., Morcuende R., Martínez-Carrasco R. (2007). Gas exchange acclimation to elevated CO_2_ in upper-sunlit and lower-shaded canopy leaves in relation to nitrogen acquisition and partitioning in wheat grown in field chambers. Environ. Exp. Bot..

[23] Ainsworth E.A., Rogers A., Nelson R., Long S.P. (2004). Testing the “source-sink” hypothesis of down-regulation of photosynthesis in elevated [CO_2_] in the field with single gene substitutions in Glycine max. Agric. For. Meteorol..

[24] Tausz-Posch S., Norton R.M., Seneweera S., Fitzgerald G.J., Tausz M. (2013). Will intra-specific differences in transpiration efficiency in wheat be maintained in a high CO_2_ world?. A FACE study. Physiol. Plant..

[25] Ziska L.H., Morris C.F., Goins E.W. (2004). Quantitative and qualitative evaluation of selected wheat varieties released since 1903 to increasing atmospheric carbon dioxide: Can yield sensitivity to carbon dioxide be a factor in wheat performance?. Glob. Chang. Biol..

[26] Ziska L.H. (2008). Three-year field evaluation of early and late 20th century spring wheat cultivars to projected increases in atmospheric carbon dioxide. F. Crop. Res..

[27] Schynder H. (1993). The role of carbohydrate storage and redistribution in the source-sink relations of wheat and barley during grain filling—A review. New Phytol..

[28] Yang J., Zhang J. (2006). Grain filling of cereals under soil drying. New Phytol..

[29] Cossani C.M., Reynolds M.P. (2012). Physiological traits for improving heat tolerance in wheat. Plant Physiol..

[30] Djanaguiraman M., Narayanan S., Erdayani E., Prasad P.V.V. (2020). Effects of high temperature stress during anthesis and grain filling periods on photosynthesis, lipids and grain yield in wheat. BMC Plant Biol..

[31] Lobell D.B., Field C.B. (2007). Global scale climate–crop yield relationships and the impacts of recent warming. Environ. Res. Lett..

[32] Farooq M., Bramley H., Palta J.A., Siddique K.H.M. (2011). Heat stress in wheat during reproductive and grain-filling phases. CRC. Crit. Rev. Plant Sci..

[33] Prasad P.V.V., Djanaguiraman M. (2014). Response of floret fertility and individual grain weight of wheat to high temperature stress: Sensitive stages and thresholds for temperature and duration. Funct. Plant Biol..

[34] Bergkamp B., Impa S.M., Asebedo A.R., Fritz A.K., Jagadish S.V.K. (2018). Prominent winter wheat varieties response to post-flowering heat stress under controlled chambers and field based heat tents. F. Crop. Res..

[35] Barnabás B., Jäger K., Fehér A. (2008). The effect of drought and heat stress on reproductive processes in cereals. Plant Cell Environ..

[36] Dupont F.M., Hurkman W.J., Vensel W.H., Tanaka C., Kothari K.M., Chung O.K., Altenbach S.B. (2006). Protein accumulation and composition in wheat grains: Effects of mineral nutrients and high temperature. Eur. J. Agron..

[37] Asseng S., Ewert F., Martre P., Rötter R.P., Lobell D.B., Cammarano D., Kimball B.A., Ottman M.J., Wall G.W., White J.W. (2015). Rising temperatures reduce global wheat production. Nat. Clim. Chang..

[38] Liu B., Asseng S., Müller C., Ewert F., Elliott J., Lobell D.B., Martre P., Ruane A.C., Wallach D., Jones J.W. (2016). Similar estimates of temperature impacts on global wheat yield by three independent methods. Nat. Clim. Chang..

[39] Hatfield J.L., Dold C. (2018). Agroclimatology and wheat production: Coping with climate change. Front. Plant Sci..

[40] Fitzgerald G.J., Tausz M., O’Leary G., Mollah M.R., Tausz-Posch S., Seneweera S., Mock I., Löw M., Partington D.L., Mcneil D. (2016). Elevated atmospheric [CO_2_] can dramatically increase wheat yields in semi-arid environments and buffer against heat waves. Glob. Chang. Biol..

[41] Ainsworth E.A., Long S.P. (2021). 30 years of free-air carbon dioxide enrichment (FACE): What have we learned about future crop productivity and its potential for adaptation?. Glob. Chang. Biol..

[42] Sánchez De La Puente L., Pérez Pérez P., Martínez-Carrasco R., Morcuende Morcuende R., Martín Del Molino I.M. (2000). Action of elevated CO_2_ and high temperatures on the mineral chemical composition of two varieties of wheat. Agrochimica.

[43] Högy P., Kottmann L., Schmid I., Fangmeier A. (2019). Heat, wheat and CO_2_: The relevance of timing and the mode of temperature stress on biomass and yield. J. Agron. Crop Sci..

[44] Sabella E., Aprile A., Negro C., Nicolì F., Nutricati E., Vergine M., Luvisi A., De Bellis L. (2020). Impact of climate change on durum wheat yield. Agronomy.

[45] Marcos-Barbero E.L., Pérez P., Martínez-Carrasco R., Arellano J.B., Morcuende R. (2021). Genotypic variability on grain yield and grain nutritional quality characteristics of wheat grown under elevated CO_2_ and high temperature. Plants.

[46] Wang L., Feng Z., Schjoerring J.K. (2013). Effects of elevated atmospheric CO2 on physiology and yield of wheat (Triticum aestivum L.): A meta-analytic test of current hypotheses. Agric. Ecosyst. Environ.

[47] Erice G., Sanz-Sáez Á., González-Torralba J., Méndez-Espinoza A.M., Urretavizcaya I., Nieto M.T., Serret M.D., Araus J.L., Irigoyen J.J., Aranjuelo I. (2019). Impact of elevated CO_2_ and drought on yield and quality traits of a historical (Blanqueta) and a modern (Sula) durum wheat. J. Cereal Sci..

[48] Tausz-Posch S., Seneweera S., Norton R.M., Fitzgerald G.J., Tausz M. (2012). Can a wheat cultivar with high transpiration efficiency maintain its yield advantage over a near-isogenic cultivar under elevated CO_2_?. F. Crop. Res..

[49] Högy P., Keck M., Niehaus K., Franzaring J., Fangmeier A. (2010). Effects of atmospheric CO_2_ enrichment on biomass, yield and low molecular weight metabolites in wheat grain. J. Cereal Sci..

[50] Tausz-Posch S., Dempsey R.W., Seneweera S., Norton R.M., Fitzgerald G., Tausz M. (2015). Does a freely tillering wheat cultivar benefit more from elevated CO_2_ than a restricted tillering cultivar in a water-limited environment?. Eur. J. Agron..

[51] Bourgault M., Dreccer M.F., James A.T., Chapman S.C. (2013). Genotypic variability in the response to elevated CO_2_ of wheat lines differing in adaptive traits. Funct. Plant Biol..

[52] Tausz M., Tausz-Posch S., Norton R.M., Fitzgerald G.J., Nicolas M.E., Seneweera S. (2013). Understanding crop physiology to select breeding targets and improve crop management under increasing atmospheric CO_2_ concentrations. Environ. Exp. Bot..

[53] Makino A., Tadahiko M. (1999). Photosynthesis and plant growth at elevated levels of CO_2_. Plant Cell Physiol..

[54] Högy P., Brunnbauer M., Koehler P., Schwadorf K., Breuer J., Franzaring J., Zhunusbayeva D., Fangmeier A. (2013). Grain quality characteristics of spring wheat (Triticum aestivum) as affected by free-air CO_2_ enrichment. Environ. Exp. Bot..

[55] Panozzo J.F., Walker C.K., Partington D.L., Neumann N.C., Tausz M., Seneweera S., Fitzgerald G.J. (2014). Elevated carbon dioxide changes grain protein concentration and composition and compromises baking quality. A FACE study. J. Cereal Sci..

[56] Högy P., Fangmeier A. (2008). Effects of elevated atmospheric CO_2_ on grain quality of wheat. J. Cereal Sci..

[57] Fernando N., Panozzo J., Tausz M., Norton R.M., Neumann N., Fitzgerald G.J., Seneweera S. (2014). Elevated CO_2_ alters grain quality of two bread wheat cultivars grown under different environmental conditions. Agric. Ecosyst. Environ..

[58] Nuttall J.G., O’Leary G.J., Panozzo J.F., Walker C.K., Barlow K.M., Fitzgerald G.J. (2017). Models of grain quality in wheat—A review. F. Crop. Res..

[59] Tashiro T., Wardlaw I. (1990). The response to high temperature shock and humidity changes prior to and during the early stages of grain development in wheat. Funct. Plant Biol..

[60] Wollenweber B., Porter J.R., Schellberg J. (2003). Lack of interaction between extreme high-temperature events at vegetative and reproductive growth stages in wheat. J. Agron. Crop Sci..

[61] Weichert H., Högy P., Mora-Ramirez I., Fuchs J., Eggert K., Koehler P., Weschke W., Fangmeier A., Weber H. (2017). Grain yield and quality responses of wheat expressing a barley sucrose transporter to combined climate change factors. J. Exp. Bot..

[62] González F.G., Slafer G.A., Miralles D.J. (2005). Floret development and survival in wheat plants exposed to contrasting photoperiod and radiation environments during stem elongation. Funct. Plant Biol..

[63] Foulkes M.J., Slafer G.A., Davies W.J., Berry P.M., Sylvester-Bradley R., Martre P., Calderini D.F., Griffiths S., Reynolds M.P. (2011). Raising yield potential of wheat. III. Optimizing partitioning to grain while maintaining lodging resistance. J. Exp. Bot..

[64] Waddington S.R., Ransom J.K., Osmanzai M., Saunders D.A. (1986). Improvement in the yield potential of bread wheat adapted to northwest mexico 1. Crop Sci..

[65] Acreche M.M., Slafer G.A. (2006). Grain weight response to increases in number of grains in wheat in a Mediterranean area. F. Crop. Res..

[66] Miralles D.J., Slafer G.A. (1995). Individual grain weight responses to genetic reduction in culm length in wheat as affected by source-sink manipulations. F. Crop. Res..

[67] Sayre K.D., Rajaram S., Fischer R.A. (1997). Yield potential progress in short bread wheats in northwest Mexico. Crop Sci..

[68] Shearman V.J., Sylvester-Bradley R., Scott R.K., Foulkes M.J. (2005). Physiological processes associated with wheat yield progress in the UK. Crop Sci..

[69] Xiao Y.G., Qian Z.G., Wu K., Liu J.J., Xia X.C., Ji W.Q., He Z.H. (2012). Genetic gains in grain yield and physiological traits of winter wheat in shandong province, china, from 1969 to 2006. Crop Sci..

[70] Sanchez-Garcia M., Royo C., Aparicio N., Martín-Sánchez J.A., Álvaro F. (2013). Genetic improvement of bread wheat yield and associated traits in Spain during the 20th century. J. Agric. Sci..

[71] Miralles D.J., Katz S.D., Colloca A., Slafer G.A. (1998). Floret development in near isogenic wheat lines differing in plant height. F. Crop. Res..

[72] Canevara M.G., Romani M., Corbellini M., Perenzin M., Borghi B. (1994). Evolutionary trends in morphological, physiological, agronomical and qualitative traits of Triticum aestivum L. cultivars bred in Italy since 1900. Eur. J. Agron..

[73] Royo C., Briceño-Felix G.A., Bojean A.P., Angus W.J., van Ginkel M. (2011). Spanish wheat pool. The World Wheat Book: A History of Wheat Breeding.

[74] Araus J.L., Slafer G.A., Royo C., Serret M.D. (2008). Breeding for yield potential and stress adaptation in cereals. CRC. Crit. Rev. Plant Sci..

[75] Dreccer M.F., Chapman S.C., Rattey A.R., Neal J., Song Y., Christopher J.T., Reynolds M. (2013). Developmental and growth controls of tillering and water-soluble carbohydrate accumulation in contrasting wheat (*Triticum aestivum* L.) genotypes: Can we dissect them?. J. Exp. Bot..

[76] Duggan B.L., Richards R.A., van Herwaarden A.F., Fettell N.A. (2005). Agronomic evaluation of a tiller inhibition gene (tin) in wheat. I. Effect on yield, yield components, and grain protein. Aust. J. Agric. Res..

[77] Reynolds M., Foulkes M.J., Slafer G.A., Berry P., Parry M.A.J., Snape J.W., Angus W.J. (2009). Raising yield potential in wheat. J. Exp. Bot..

[78] Lopes M.S., Reynolds M.P., Manes Y., Singh R.P., Crossa J., Braun H.J. (2012). Genetic yield gains and changes in associated traits of CIMMYT spring bread wheat in a “Historic” set representing 30 years of breeding. Crop Sci..

[79] Morgounov A., Zykin V., Belan I., Roseeva L., Zelenskiy Y., Gomez-Becerra H.F., Budak H., Bekes F. (2010). Genetic gains for grain yield in high latitude spring wheat grown in Western Siberia in 1900–2008. F. Crop. Res..

[80] Zheng T.C., Zhang X.K., Yin G.H., Wang L.N., Han Y.L., Chen L., Huang F., Tang J.W., Xia X.C., He Z.H. (2011). Genetic gains in grain yield, net photosynthesis and stomatal conductance achieved in Henan Province of China between 1981 and 2008. F. Crop. Res..

[81] Del Pozo A., Matus I., Serret M.D., Araus J.L. (2014). Agronomic and physiological traits associated with breeding advances of wheat under high-productive Mediterranean conditions. The case of Chile. Environ. Exp. Bot..

[82] Mondal S., Dutta S., Crespo-Herrera L., Huerta-Espino J., Braun H.J., Singh R.P. (2020). Fifty years of semi-dwarf spring wheat breeding at CIMMYT: Grain yield progress in optimum, drought and heat stress environments. F. Crop. Res..

[83] Gutiérrez E., Gutiérrez D., Morcuende R., Verdejo A.L., Kostadinova S., Martinez-Carrasco R., Pérez P. (2009). Changes in leaf morphology and composition with future increases in CO_2_ and temperature revisited: Wheat in field chambers. J. Plant Growth Regul..

[84] Gutiérrez D., Morcuende R., Del Pozo A., Martínez-Carrasco R., Pérez P. (2013). Involvement of nitrogen and cytokinins in photosynthetic acclimation to elevated CO_2_ of spring wheat. J. Plant Physiol..

[85] Gourdji S.M., Mathews K.L., Reynolds M., Crossa J., Lobell D.B. (2013). An assessment of wheat yield sensitivity and breeding gains in hot environments. Proc. R. Soc. B Biol. Sci..

[86] Córdoba J., Molina-Cano J.L., Pérez P., Morcuende R., Moralejo M., Savé R., Martínez-Carrasco R. (2015). Photosynthesis-dependent/independent control of stomatal responses to CO_2_ in mutant barley with surplus electron transport capacity and reduced SLAH3 anion channel transcript. Plant Sci..

[87] Vicente R., Pérez P., Martínez-Carrasco R., Gutiérrez E., Morcuende R. (2015). Nitrate supply and plant development influence nitrogen uptake and allocation under elevated CO_2_ in durum wheat grown hydroponically. Acta Physiol. Plant..

[88] Vicente R., Pérez P., Martínez-Carrasco R., Feil R., Lunn J.E., Watanabe M., Arrivault S., Stitt M., Hoefgen R., Morcuende R. (2016). Metabolic and transcriptional analysis of durum wheat responses to elevated CO_2_ at low and high nitrate supply. Plant Cell Physiol..

[89] (2019). R Core Team R: A Language and Environment for Statistical Computing.

[90] Revelle W. Package “psych”—Procedures for Psychological, Psychometric and Personality Research. R Package Version 2.1.3. https://cran.r-project.org/package=psych.

[91] Husson F., Josse J., Pages J. (2010). Principal component methods-hierarchical clustering-partitional clustering: Why would we need to choose for visualizing data. Appl. Math. Dep..

[92] Signorell A. DescTools: Tools for Descriptive Statistics. R package version 0.99.42. https://cran.r-project.org/package=DescTools.

[93] Wickham H. (2007). Reshaping Data with the reshape Package. J. Stat. Softw..

[94] Shannon P. (2003). Cytoscape: A software environment for integrated models of biomolecular interaction networks. Genome Res..

