# Supplementary material for: Screening for Higher Grain Yield and Biomass among Sixty Bread Wheat Genotypes Grown under Elevated CO2 and High-Temperature Conditions
Source: Plants (Basel). 2021 Aug 3;10(8):1596. doi: 10.3390/plants10081596 (PMC8401911; doi:10.3390/plants10081596)
Supplement: Supplementary file 1 [file plants-10-01596-s001.zip › plants-1323446-supplementary.pdf]

plants MDPI

Table S1. Correlation coefficient matrix for wheat production and grain yield components among sixty wheat genotypes grown under elevated CO_2 and high temperature.

	**Stalk**	**Chaff**	**Grain yield**	**Grain number**	**Ear number**	**Grain weight**	**GYE**	**GNE**	**HI**	**Height**
**Aboveground**	**0.90**	**0.77**	**0.88**	**0.76**	**0.57**	-0.11	**0.31**	**0.31**	0.00	**0.15**
**Stalk**		**0.60**	**0.64**	**0.57**	**0.47**	-0.10	**0.15**	**0.19**	**-0.31**	**0.28**
**Chaff**			**0.60**	**0.51**	**0.47**	0.00	**0.16**	0.11	**-0.15**	0.07
**Grain yield**				**0.85**	**0.54**	-0.11	**0.45**	**0.43**	**0.42**	0.03
**Grain number**					**0.54**	**-0.58**	**0.32**	**0.61**	**0.35**	-0.06
**Ear number**						**-0.16**	**-0.44**	**-0.29**	0.10	0.02
**Grain weight**							0.05	**-0.54**	-0.02	**0.19**
**GYE**								**0.78**	**0.37**	0.00
**GNE**									**0.32**	-0.10
**HI**										**-0.27**

GNE: grain number ear^{-1}; *GYE*: grain yield ear^{-1}; *HI*: harvest index.

Data was generated from Spearman correlation analysis. Values in bold represent significance ($p < 0.05$).

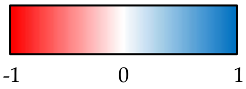

Table S2. Average days from sowing to ear emergence, anthesis and maturity developmental stages among sixty wheat genotypes grown under elevated CO_2 and high temperature.

Genotype	Ee	Anthesis	Maturity	Genotype	Ee	Anthesis	Maturity	Genotype	Ee	Anthesis	Maturity
**150**	58	60	95	**16**	64	65	99	**35**	57	60	95
**74**	65	66	99	**22**	63	65	98	**4**	60	62	96
**23**	68	69	102	**13**	63	64	95	**26**	59	61	95
**8**	65	66	97	**92**	66	67	102	**48**	59	62	96
**76**	64	65	99	**117**	62	64	93	**99**	65	65	96
**15**	60	63	97	**79**	61	63	96	**53**	57	59	94
**73**	61	63	95	**33**	59	61	96	**59**	61	63	97
**21**	64	66	101	**69**	64	65	98	**83**	66	67	102
**9**	65	66	97	**31**	57	61	95	**10**	63	64	98
**118**	57	60	92	**19**	66	66	100	**65**	61	63	94
**81**	63	65	97	**114**	62	63	93	**42**	61	63	95
**77**	65	66	99	**97**	62	63	94	**107**	61	63	96
**115**	57	60	92	**113**	66	67	97	**11**	60	62	96
**87**	65	66	99	**25**	60	62	96	**58**	65	66	100
**29**	60	62	99	**18**	59	61	93	**5**	58	60	93
**46**	63	64	95	**70**	66	67	102	**43**	61	62	98
**119**	63	65	96	**63**	60	61	94	**94**	63	65	97
**6**	60	62	97	**105**	63	65	98	**95**	58	60	93
**110**	64	65	98	**88**	65	66	99	**61**	62	64	95
**71**	62	64	96	**93**	60	61	94	**41**	59	61	96

Ee: Ear emergence; *GNE*: grain number ear^{-1}; *GYE*: grain yield ear^{-1}; *HI*: harvest index.

Data was generated from Spearman correlation analysis. Values in bold represent significance ($p < 0.05$).

Minimum Maximum

Table S3. Correlation coefficient matrix for average wheat production and grain yield components with days from sowing to ear emergence, anthesis and maturity developmental stages among sixty wheat genotypes grown under elevated CO_2 and high temperature.

	Ear emergence	Anthesis	Maturity
**Aboveground**	-0,25	**-0,29**	**-0,33**
**Stalk**	-0,12	-0,16	**-0,31**
**Chaff**	**-0,37**	**-0,42**	**-0,40**
**Grain yield**	-0,24	-0,28	-0,22
**Grain number**	-0,09	-0,13	-0,10
**Ear number**	**-0,30**	**-0,30**	**-0,35**
**Grain weight**	-0,08	-0,03	-0,03
**GYE**	0,11	0,07	0,18
**GNE**	0,14	0,09	0,19
**HI**	-0,04	-0,01	0,23
**Height**	0,21	0,21	-0,13

GNE: grain number ear^{-1}; *GYE*: grain yield ear^{-1}; *HI*: harvest index.

Data was generated from Spearman correlation analysis. Values in bold represent significance ($p < 0.05$).

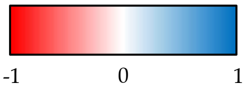

Table S4. Catalogue of the 60 wheat genotypes employed in the present study. Pedigree, accession code and top name of genotypes belonging to the Heat Tolerance Wheat Screening Nursery (8TH HTWSN) collection of the International Maize and Wheat Improvement Centre (CIMMYT) are provided.

Cultivar	Genotype	Pedigree	Accesion	Reception year	Top Name
SUPER 152	4	PFAU/SERI.1B//AMADINA/3/WAXWING	BW 43354	2009	CGSS-02-Y-00153S-099M-099Y-099M-46Y-0B
BAJ #1	5	WAXWING/4/SONOITA F 81/TRAP #1/3/KAUZ*2/TRAP//KAUZ	BW 42811	2009	CGSS01Y00134S-099Y-099M-099M-13Y-0B
REEDLING #1	6	-	-	-	CMSS06Y00605T-099TOPM-099Y-099ZTM-099Y-099M-11WGY-0B
	8	KACHU/KIRITATI	BW 49924	2012	CMSS07Y00127S-0B-099Y-099M-099NJ-099NJ-6WGY-0B
	9	KACHU #1//WEEBILL1*2/KUKUNA	BW 49925	2013	CMSS07Y00129S-0B-099Y-099M-099NJ-099NJ-14WGY-0B
	10	KIRITATI/WEEBILL1//FRANCOLIN #1	BW 49926	2012	CMSS07Y00174S-0B-099Y-099M-099Y-5M-0WGY
	11	SUPER 152/BAJ #1	BW 49927	2012	CMSS07Y00195S-0B-099Y-099M-099Y-16M-0WGY
	13	BAJ #1/3/KIRITATI//ATTILA*2/PASTOR	BW 49930	2012	CMSS07Y00288S-0B-099Y-099M-099Y-17M-0WGY
	15	SUPER 152/AKURI//SUPER 152	BW 49936	2012	CMSS07Y00965T-099TOPM-099Y-099M-099Y-23M-0WGY
	16	BECARD/FRANCOLIN	BW 49948	2012	CMSS07B00235S-099M-099Y-099M-25WGY-0B
	18	BECARD//ND643/2*WEEBILL1	BW 49950	2012	CMSS07B00377S-099M-099NJ-099NJ-23WGY-0B
	19	FRET2*2/BRAMBLING//BECARD/3/WEEBILL1*2/BRAMBLING	BW 49952	2013	CMSS07B00560T-099TOPY-099M-099Y-099M-19WGY-0B
	21	KACHU/BECARD//WEEBILL1*2/BRAMBLING	BW 49954	2012	CMSS07B00580T-099TOPY-099M-099Y-099M-10WGY-0B
	22	KAUZ/PASTOR//PBW343/3/KIRITATI/4/FRANCOLIN	BW 49955	2012	CMSS07B00585T-099TOPY-099M-099Y-099M-11WGY-0B
	23	SUPER 152*2/TECUE #1	BW 49956	2012	CMSS07B00614T-099TOPY-099M-099Y-099M-49WGY-0B
	25	WEEBILL1*2/4/SONOITA F 81/TRAP #1/3/KAUZ*2/TRAP//KAUZ/5/BAJ #1	BW 49388	2012	CMSS06B00162S-0Y-099ZTM-099Y-099M-7WGY-0B
	26	NACOZARI F 76/TH.ACUTUM//3*PAVON F76/3/MIRLO/BUCKBUCK/4/ 2*PASTOR/ 5/T.DICOCCON PI94624/AE.SQUARROSA (409)//BACANORA T 88/ 6/WEEBILL4// BABAX.1B.1B*2/PARULA/3/PASTOR	BW 50018	2013	CMSS06B01043T-099TOPY-099Y-39M-0Y-2B-0Y
	29	CHIBIA//PARULA II/CM65531/3/SUPER KAUZ/BAVIACORA M 92/4/MUNAL #1	BW 50026	2013	CMSS07Y00066S-0B-099Y-099M-099Y-43M-0WGY
	31	FRET2/TUKURU//FRET2/3/MUNAL #1	BW 50028	2012	CMSS07Y00093S-0B-099Y-099M-099NJ-099NJ-9WGY-0B
	33	KACHU #1//WEEBILL1*2/KUKUNA	BW 50036	2013	CMSS07Y00129S-0B-099Y-099M-099NJ-099NJ-12WGY-0B
	35	KACHU//KIRITATI/WEEBILL1	BW 50038	2013	CMSS07Y00130S-0B-099Y-099M-099NJ-099NJ-8WGY-0B
	41	SUPER 152/BAJ #1	BW 50048	2013	CMSS07Y00195S-0B-099Y-099M-099Y-5M-0WGY
	42	SUPER 152/BAJ #1	BW 50049	2012	CMSS07Y00195S-0B-099Y-099M-099Y-25M-0WGY
	43	SUPER 152//WEEBILL1*2/BRAMBLING	BW 50050	2012	CMSS07Y00196S-0B-099Y-099M-099Y-6M-0WGY
	46	BAJ #1/3/KIRITATI//ATTILA*2/PASTOR	BW 50059	2012	CMSS07Y00288S-0B-099Y-099M-099Y-3M-0WGY
	48	WHEATEAR*2/3/FRET2/WEEBILL1//TACUPETO F2001	BW 50085	2012	CMSS07Y00794T-099TOPM-099Y-099M-099Y-3M-0WGY
	53	SUPER 152*2/MUU	BW 50107	2012	CMSS07Y00964T-099TOPM-099Y-099M-099Y-8M-0WGY
	58	WEEBILL1*2/BRAMBLING//JUCHI F2000/3/WEEBILL1*2/BRAMBLING	BW 50117	2013	CMSS07Y01044T-099TOPM-099Y-099M-099Y-6M-0WGY
	59	WEEBILL1/KUKUNA//TACUPETO F2001*2/6/PAVON F 76//CARIANCA 422/ ANAHUAC F 75/5/BOBWHITE/CROW//BUCKBUCK/PAVON F 76/3/YECORA F 70/4/TRAP #1	BW 50119	2012	CMSS07Y01070T-099TOPM-099Y-099M-099Y-20M-0WGY
	61	TOBARITO M 97/PASTOR*2//AKURI	BW 50122	2013	CMSS07Y01094T-099TOPM-099Y-099M-099NJ-099NJ-17WGY-0B
	63	INQALAB 91*2/KUKUNA*2//HUIRIVIS #1	BW 50126	2012	CMSS07Y01115T-099TOPM-099Y-099M-099Y-9M-0WGY

Table S4. Continuation

	65	SUPER 152/VILLA JUAREZ F2009	BW 50150	2013	CMSS07B00144S-099M-099Y-099M-5WGY-0B
	69	BECARD/FRANCOLIN	BW 50181	2012	CMSS07B00235S-099M-099Y-099M-8WGY-0B
	70	BECARD/CHYAKHURA	BW 50184	2013	CMSS07B00236S-099M-099Y-099M-13WGY-0B
	71	WEEBILL1*2/KUKUNA/4/WHEATEAR/KUKUNA/3/C80.1/3*BATAVIA//2*WEEBILL1	BW 50185	2012	CMSS07B00240S-099M-099Y-099M-12WGY-0B
	73	WEEBILL1/KUKUNA//TACUPETO F2001/4/WHEATEAR/KUKUNA/3/C80.1/ 3*BATAVIA//2*WEEBILL1	BW 50191	2012	CMSS07B00245S-099M-099Y-099M-10WGY-0B
	74	WEEBILL1/KUKUNA//TACUPETO F2001/3/QUAIU #2	BW 50193	2012	CMSS07B00246S-099M-099Y-099M-5WGY-0B
	76	WHEATEAR/KUKUNA/3/C80.1/3*BATAVIA//2*WEEBILL1/4/QUAIU	BW 50196	2012	CMSS07B00264S-099M-099NJ-099NJ-2WGY-0B
	77	WHEATEAR/KUKUNA/3/C80.1/3*BATAVIA//2*WEEBILL1/4/QUAIU	BW 50197	2012	CMSS07B00264S-099M-099NJ-099NJ-7WGY-0B
	79	SUPER 152/CHYAKHURA #1	BW 50208	2013	CMSS07B00339S-099M-099Y-099M-11WGY-0B
	81	PBW 65/2*PASTOR/3/KIRITATI//PBW 65/2*SERI.1B/4/DANPHE #1	BW 50215	2012	CMSS07B00513T-099TOPY-099M-099Y-099M-12WGY-0B
	83	CIANO T 79//PF70354/MUSALA/3/PASTOR/4/BAVIACORA M 92/5/KIRITATI// ATTILA*2/PASTOR/6/CHYAKHURA	BW 50229	2013	CMSS07B00550T-099TOPY-099M-099Y-099M-6WGY-0B
	87	KACHU/BECARD//WEEBILL1*2/BRAMBLING	BW 50238	2013	CMSS07B00580T-099TOPY-099M-099NJ-099NJ-32WGY-0B
	88	KACHU/BECARD//WEEBILL1*2/BRAMBLING	BW 50239	2013	CMSS07B00580T-099TOPY-099M-099NJ-099NJ-34WGY-0B
	92	SUPER 152*2/TECUE #1	BW 50248	2013	CMSS07B00614T-099TOPY-099M-099Y-099M-16WGY-0B
	93	WEEBILL1*2/KUKUNA//KIRITATI/3/WEEBILL1*2/KUKUNA	BW 50263	2013	CMSS07B00682T-099TOPY-099M-099Y-099M-22WGY-0B
	94	WEEBILL1*2/KURUKU*2//SUPER 152	BW 50264	2013	CMSS07B00685T-099TOPY-099M-099Y-099M-17WGY-0B
	95	FRET2/KUKUNA//FRET2/3/HEILO/4/BLOUK #1	BW 50266	2013	CMSS07B00715T-099TOPY-099M-099Y-099M-7WGY-0B
	97	ND643/2*WEEBILL1//ATTILA*2/PBW 65/3/MUNAL	BW 50270	2012	CMSS07B00807T-099TOPY-099M-099NJ-099NJ-1WGY-0B
	99	ND643//2*ATTILA*2/PASTOR/3/WEEBILL1*2/KURUKU/4/WEEBILL1*2/BRAMBLING	BW 50276	2012	CMSS07B00833T-099TOPY-099M-099NJ-099NJ-3WGY-0B
	105	REH/HARE//2*BACANORA T 88/3/CROC_1/AE.SQUARROSA (213)//PAPAGO M 86/ 4/HUITES F 95/5/T.DICOCCON PI94624/AE.SQUARROSA (409)//BACANORA T 88/ 6/REH/HARE//2*BACANORA T 88/3/CROC_1/AE.SQUARROSA (213)//PAPAGO M 86/4/HUITES F 95/7/MUTUS	BW 49979	2013	CMSS07Y01305T-099Y-43M-0Y-2B-0Y
	107	BLOUK #1//TACUPETO F2001*2/KIRITATI	BW 50284	2013	CMSS07Y00036S-0B-099Y-099M-099NJ-099NJ-1RGY-0B
	110	QUAIU #3//TACUPETO F2001*2/KIRITATI	BW 50287	2013	CMSS07Y00040S-0B-099Y-099M-099NJ-099NJ-5RGY-0B
	113	FRANCOLIN #1/2*BLOUK #1	BW 50296	2013	CMSS07Y00670T-099TOPM-099Y-099M-099Y-11M-0RGY
	114	PFAU/WEAVER*2//TUKURU/3/BLOUK #1/4/QUAIU	BW 50298	2013	CMSS07Y00695T-099TOPM-099Y-099M-099Y-11M-0RGY
	115	VOROBEY/FISCAL//KACHU/3/WEEBILL1*2/BRAMBLING	BW 50299	2013	CMSS07Y00706T-099TOPM-099Y-099M-099Y-11M-0RGY
	117	WEEBILL1*2/BRAMBLING/5/WEEBILL1*2/4/SONOITA F 81/TRAP #1/3/KAUZ*2/ TRAP//KAUZ/6/WEEBILL1*2/BRAMBLING	BW 50303	2013	CMSS07Y00815T-099TOPM-099Y-099M-099Y-9M-0RGY
	118	BLOUK #1/MUNAL	BW 50308	2013	CMSS07B00024S-099M-099Y-099M-21RGY-0B
	119	BECARD/3/PASTOR//MUNIA/ALTAR 84	BW 50313	2013	CMSS07B00468S-099M-099Y-099M-18RGY-0B
GAZUL	150		20351	1992	

Table S5. Statistical design with tests employed, assessed assumptions for each test, central tendency estimator used and indices for the effect size.

	One-group comparison		Multiple group comparison: Independent measures			
	**Normal distribution**	**Non-normal distribution**	**Normal distribution**		**Non-normal distribution**	
**Assumptions**	-	-	**Homogeneity of variances**	**Heterogeneity of variances**	**Homogeneity of variances**	**Heterogeneity of variances**
	-	-	Unbalanced groups	Un/balanced groups	Un/balanced groups	Un/balanced groups
	Absence of extreme outliers	Absence of extreme outliers	Absence of extreme outliers	Absence of extreme outliers	Dealing with extreme outliers	Dealing with extreme outliers
**Type of analysis**	**T test**	**Wilcoxon test**	**ANOVA**	**ANOVA with heteroscedasticity correction**	**Kruskal-Wallis (K-W) test**	**Robust ANOVA for medians**
**R function**	*t_test* [1]	*wilcox_test* [1]	*anova_test* [1]	*anova_test* [1]	*Kruskal_test* [1]	*med1way* [2]
**Post-hoc test**	-	-	**Pairwise T test**	**Pairwise T test**	**Wilcox**	**Pairwise permuta-tion tests for percentiles**
**R function**	-	-	*pairwise.t.test* [3]	*pairwise.t.test* [3]	*pairwise.wilcox.test* [1]	*pairwisePercentileTest* [4]
**Central tendency estimator**	*Mean*	*Trimmed mean (20%)*	*Mean*	*Mean*	*Median*	
**Effect size** – Indices	d_{Cohen}		η^2	η^2	ω^2	
**Effect size** – R function	*cohens_d* [1]		*Included in anova_test* [1]	*eta.F* [5]	*omega.F* [5]	
**Effect size** – Interpretation – No effect	d < 0.20		$\eta^2 < 0.01$		$\omega^2 < 0.01$	
**Effect size** – Interpretation – Small	0.20 ≤ d < 0.50		$0.01 \leq \eta^2 > 0.06$		$0.01 \leq \omega^2 > 0.06$	
**Effect size** – Interpretation – Medium	0.50 ≤ d < 0.80		$0.06 \leq \eta^2 > 0.14$		$0.06 \leq \omega^2 > 0.14$	
**Effect size** – Interpretation – Large	d ≥ 0.80		$\eta^2 \geq 0.14$		$\omega^2 \geq 0.14$	

Note*: The Benjamini, Hochberg, and Yekutieli method for the false discovery rate control was applied for pairwise comparisons (*pairwise.t.test* [3]), with corrections for homo/heterogeneity of variances.

Other references employed to perform this table:

- Selection of tests: [6–10]
- Effect size: [7,11–15]

References

1. Kassambara, A. rstatix: Pipe-friendly framework for basic statistical tests 2021.
2. Mair, P.; Schoenbrodt, F.; Wilcox, R. WRS2: Wilcox robust estimation and testing 2016.
3. Team R Development Core A Language and Environment for Statistical Computing. *R Found. Stat. Comput.* 2018, *2*, https://www.R-project.org.
4. Mangiafico, S. rcompanion: Functions to Support Extension Education Program Evaluation 2020.
5. Buchanan, E.M.; Gillenwaters, A.; Scofield, J.E.; Valentine, K.D. MOTE: Measure of the Effect: Package to assist in effect size calculations and their confidence intervals 2019.
6. Kassambara, A. *Practical Statistics in R II - Comparing Groups: Numerical Variables*; Datanovia, 2019;
7. Mair, P.; Wilcox, R.; Patil, I. Robust Statistical Methods Using WRS2 (vignette) 2021, 41.
8. García-Pérez, A. Métodos avanzados de estadística aplicada. Métodos robustos y de remuestreo. **2005**, 256.
9. Mair, P.; Wilcox, R. Robust Statistical Methods Using WRS2. *Behav. Res. Methods* **2020**, *52*, 464–488, doi:10.3758/s13428-019-01246-w.
10. Mangiafico, S.S. *Summary and analysis of extension program evaluation in R*; 2016;
11. Ben-Shachar, M.; Lüdecke, D.; Makowski, D. effectsize: Estimation of Effect Size Indices and Standardized Parameters. *J. Open Source Softw.* **2020**, *5*, 2815, doi::10.21105/joss.01412.
12. Sullivan, G.M.; Feinn, R. Using Effect Size—or Why the P Value Is Not Enough . *J. Grad. Med. Educ.* **2012**, *4*, 279–282, doi:10.4300/jgme-d-12-00156.1.
13. Kotrlik, J.; Williams, H.; Jabor, K. Reporting and interpreting Effect Size in quantitative agricultural education research. *J. Agric. Educ.* **2011**, *52*, 132–142, doi:10.5032/jae.2011.01132.
14. Ellis, P.D. *The essential guide to Effect Sizes: Statistical power, meta-analysis, and the interpretation of research results*; Cambridge University Press: Cambridge, 2010; ISBN 9780511761676.
15. Brydges, C.R. Effect Size guidelines, sample size calculations, and statistical power in gerontology. *Innov. Aging* **2019**, *3*, 1–8, doi:10.1093/geroni/igz036.
